# Participatory Interventions for Sexual Health Promotion for Adolescents and Young Adults on the Internet: Systematic Review

**DOI:** 10.2196/15378

**Published:** 2020-07-31

**Authors:** Philippe Martin, Lorraine Cousin, Serge Gottot, Aurelie Bourmaud, Elise de La Rochebrochard, Corinne Alberti

**Affiliations:** 1 Université de Paris ECEVE INSERM Paris France; 2 Institut National d’Etudes Démographiques UR14 – Sexual and Reproductive Health and Rights Paris France; 3 Université Paris-Saclay, Université Paris-Sud, UVSQ CESP INSERM Le Kremlin Bicetre France; 4 GDID Santé Paris France

**Keywords:** sexual health, health promotion, internet, participatory interventions, adolescents and young adults, methods

## Abstract

**Background:**

The World Health Organization recommends the development of participatory sexuality education. In health promotion, web-based participatory interventions have great potential in view of the internet’s popularity among young people.

**Objective:**

The aim of this review is to describe existing published studies on online participatory intervention methods used to promote the sexual health of adolescents and young adults.

**Methods:**

We conducted a systematic review based on international scientific and grey literature. We used the PubMed search engine and Aurore database for the search. Articles were included if they reported studies on participatory intervention, included the theme of sexual health, were conducted on the internet (website, social media, online gaming system), targeted populations aged between 10 and 24 years, and had design, implementation, and evaluation methods available. We analyzed the intervention content, study implementation, and evaluation methods for all selected articles.

**Results:**

A total of 60 articles were included, which described 37 interventions; several articles were published about the same intervention. Process results were published in many articles (n=40), in contrast to effectiveness results (n=23). Many of the 37 interventions were developed on websites (n=20). The second most used medium is online social networks (n=13), with Facebook dominating this group (n=8). Online peer interaction is the most common participatory component promoted by interventions (n=23), followed by interaction with a professional (n=16). Another participatory component is game-type activity (n=10). Videos were broadcast for more than half of the interventions (n=20). In total, 43% (n=16) of the interventions were based on a theoretical model, with many using the Information-Motivation-Behavioral Skills model (n=7). Less than half of the interventions have been evaluated for effectiveness (n=17), while one-third (n=12) reported plans to do so and one-fifth (n=8) did not indicate any plan for effectiveness evaluation. The randomized controlled trial is the most widely used study design (n=16). Among the outcomes (evaluated or planned for evaluation), sexual behaviors are the most evaluated (n=14), followed by condom use (n=11), and sexual health knowledge (n=8).

**Conclusions:**

Participatory online interventions for young people’s sexual health have shown their feasibility, practical interest, and attractiveness, but their effectiveness has not yet been sufficiently evaluated. Online peer interaction, the major participatory component, is not sufficiently conceptualized and defined as a determinant of change or theoretical model component. One potential development would be to build a conceptual model integrating online peer interaction and support as a component.

## Introduction

Adolescent sexual exposure is of concern due to the risk of contracting sexually transmitted infections (STIs), experiencing an unwanted pregnancy, and unexpected paternity/maternity [1]. Among the 333 million new cases of STIs each year, the highest rates occur among those aged 20 to 24 years, followed by those aged 15 to 19 years [2]. Among a group of 21 countries, the pregnancy rate among those aged 15 to 19 years is highest in the United States (57 pregnancies per 1000 females) [3]. The proportion of teenage pregnancies that result in abortion varies by country, but in half of those for which recent information is available (mainly in Europe, North America, and Oceania), 35%-55% of pregnancies ended in abortion [3]. In 2014, in the United States, females aged <15 years and 15 to 19 years accounted for 0.3% and 10.4% of all reported abortions in the country, respectively [4].

Adolescence and the transition to adulthood marks the entry into sexuality. Sexual health requires a positive and respectful approach to sexuality and sexual relations, and the ability to have enjoyable and safe sexual experiences that are free from coercion, discrimination, and violence [5]. Adolescents and young adults (AYA) represent a priority population for sexual health promotion and education [6]. The associated fields of intervention encompass the development of knowledge and level of information, the development of attitudes to sexual health (attitudes toward safe sex practice, including attitudes to condom use or voluntary testing for STIs), and the development of personal competencies and supportive relational skills (critical thinking, consent, negotiation, open-mindedness, respect, self-esteem).

For example, as stated by the Information Motivation Behavioral Skills (IMB) model (applied and validated for HIV risk reduction), behavioral competencies and therefore health behaviors may be influenced by the level of information, but also by motivation, namely beliefs and attitudes toward a particular health behavior and the perceived social support (or social norm) to engage in this behavior [7]. In addition, health literacy is the ability of individuals to obtain, process, and understand the information and services necessary to make appropriate health decisions [8]. Increase health literacy would enable the improvement of appropriate health decision making with regard to sexual health, promoting equity and achieving the United Nations’ Sustainable Development Goals 2030 [9].

The recommendations of the World Health Organization are clearly stated [10]: sexuality education must be participatory (young people should not be mere passive receivers), interactive (with educators and program designers), and continuous. This education must be adapted to the language of the young people, while also teaching appropriate terminology to strengthen their communication skills.

In health promotion, digital media interventions for sexual health have great potential because of the scope and popularity of technologies such as the internet and mobile phones, especially among young people [11,12]. Interactive online interventions for sexual health promotion can also lead to better knowledge, self-efficacy, and positive sexual behavior, and have demonstrated a reduction in STIs [12].

The internet is a major health information resource, and online health information research is an important prerequisite for health empowerment and literacy [13,14]. Moreover, research on information flows and attitudes within social networks suggests that links between people can promote the exchange of relevant information between peers, and affect their attitude toward this information, as individuals are more receptive to information shared by others who are like them [15]. For example, the popularity of social networking sites and their interactive features have great potential to reach young people, and offer a new way to engage and communicate with AYAs, including the provision of appropriate education [16]. Nevertheless, their uses are for the most part “passive,” and social networking sites are not yet used as tools for multidimensional communication and networking [17].

Our research question is whether interventions for the promotion of young people's sexual health include participatory components, and if so, how they are integrated and how the interventions are evaluated. Some publications and literature reviews have investigated sexual health interventions on the internet, social media [12,18], online serious games [19], or in digital media [12,20,21]. However, no publication has focused on the participatory aspects of this type of intervention in sexual health specifically aimed at young people (participation in an activity such as online games, quizzes), particularly interactive features such as the exchange of information and experiences between peers (persons of the same age, social context, function, education, or experience) or with professionals. The aim of this review is to identify and describe existing studies and the methods used to assess online participatory interventions aimed at promoting AYA’s sexual health.

## Methods

### Overview

This systematic review was based on international scientific literature and grey literature. The review is structured in accordance with the PRISMA (Preferred Reporting Items for Systematic Reviews and Meta-Analysis) statement [22] and follows the associated guidelines (Multimedia Appendix 1). The systematic review protocol has previously been published on the PROSPERO International Prospective Registry of Technical Reviews (ID CRD42018088240).

### Inclusion Criteria

Articles were included without time restriction according to the 5 following criteria: (1) Study of an intervention including a sexual health theme; (2) Population aged between 10 and 24 years (with an average age or an interval comprising all or at least part of this age group), because the WHO defines adolescents as aged 10 to 19 years and young people as aged 15 to 24 years [23,24]; (3) Study of a participatory intervention; (4) Study of an intervention conducted on the internet (website, social media, online gaming); (5) Design, implementation, and evaluation methods must be available via the article.

### Strategy Search

The electronic search strategies are described in Multimedia Appendix 2. We used the PubMed search engine for our main search. For complementary research, we used the Aurore database of Institut National d’Études Démographiques (INED; a French public research institute), which includes scientific databases and grey literature, allowing access to a range of databases and electronic journals (see Multimedia Appendix 2 for selected international search engines). The last update was on January 28, 2019.

### Study Selection

Reports were assessed by two reviewers (PM and LC), who screened the titles and abstracts to identify relevant studies. Full texts were read when abstracts met inclusion criteria, and when abstracts were not clear enough to determine eligibility. Disagreements between reviewers were resolved by discussion. When the full text was not available, authors were contacted by email; all the contacted authors responded favorably and shared their articles with us.

### Data Collection

A standardized data collection form was developed, and two reviewers independently extracted data from studies. Our extraction grid was developed using the PICOTS (populations, interventions, comparators, outcomes, timing, and setting) elements [22], and was completed using Michie’s taxonomy [25] to collect information on the behavior change techniques (BCT) used by interventions. The studies were classified according to different types: research protocol only, effectiveness evaluation, and process evaluation. Protocol articles are planned studies containing only the conceptual and evaluative methods intended for intervention research. An effectiveness study is defined as a demonstration of an intervention’s efficacy in natural situations. It provides evidence of the intervention's effect on determinants or health outcomes. A process study provides evidence on the implementation and feasibility of an intervention, and also rates the intervention for attractiveness and acceptability. It helps to assess the reliability and quality of implementation, to clarify causal mechanisms, and to identify contextual factors associated with variations in outcomes [26].

### Analysis

For the final studies selection phase, the degree of interreader agreement was assessed for both readers through the calculation of the κ coefficient.

We conducted descriptive analyses on data collected from studies on the following points: description of the population; characteristics of study methodology; description of the intervention; description of the media used; description of methods used for effectiveness, and process evaluation. We used Michie’s [25] taxonomy to analyze the BCT used by interventions, depending on the information available in the intervention.

## Results

**Figure 1:**
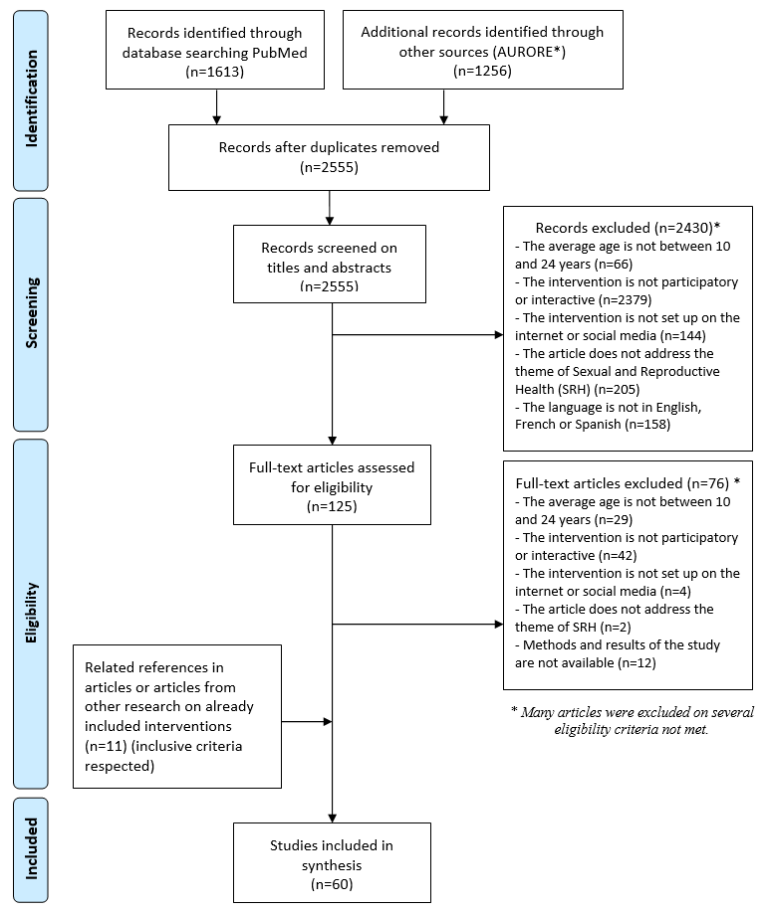
Flow chart of the literature reviewing process. Aurore is a database of Institut National d’Études Démographiques (a French public research institute) that combines scientific databases and grey literature, allowing access to a range of databases and electronic journals.

The electronic search strategies used identified a total of 2555 references after removing duplicates. After selection based on title and abstract screening, the full text of 125 references was evaluated. After this inclusion phase, 49 articles describing 37 interventions were included. For each intervention included, we searched for other publications concerning it, and 11 additional studies were included, based on the references cited in the included articles. A total of 60 articles describing 37 interventions were included; several articles were published for the same intervention (Figure 1). The degree of interreader agreement for the final selection of the 60 articles was calculated with the κ coefficient and it was equal to 0.98. All the studies included in this systematic review are available in Multimedia Appendix 3. Descriptive data for the included studies and interventions are available in Table 1. Of the 60 articles included, 52% (n=31/60) were published in the last 5 years (Table 2).

Overall, 62% of the studies (n=36/58) were conducted in the United States. Of the types of studies, 45% (n=27/60) exclusively concerned process results, 22% (n=13/60) included process results and effectiveness results, 17% (n=10/60) exclusively had effectiveness results, and 17% (n=10/60) were exclusively protocol publications. Of the 37 interventions, 51% (n=19/37) addressed sexual health holistically. Overall, 51% (n=19/37) targeted a general population. In cases where specific populations were targeted (49%, n=18/37), 44% (n=8/18) were identified by their sexual orientation. In total, 65% (n=24/37) of all interventions were for both sexes, 22% (n=8/37) were for males only, and 11% (n=4/37) were for women only. The targeted population in terms of age was mainly individuals aged 10 to 24, strictly defined in 35% of the interventions (n=13/37). However, other studies had a less specific or different range of age targeted: aged 10 to 17 years, aged 10 to >24 years, aged 18 to 24 years, or aged 18 to >24 years; some studies simply referred to “students” or “youth.” In total, 43% (n=16/37) used multiple recruitment methods.

**Table 1:** Description of the characteristics of the 60 articles and the 37 interventions.

Characteristics			Studies, n (%)
**Characteristics of articles**			
	**Year of publication (n=60)**		
		2006-2009	2 (3)
		2010-2014	27 (45)
		2015-2019	31 (52)
	**Study country (n=58; NI^a^=2)**		
		United States	36 (62)
		Canada	1 (2)
		United Kingdom	4 (7)
		Netherlands	1 (2)
		Europe (other)	2 (3)
		Australia	3 (5)
		Uganda	4 (7)
		Brazil	2 (3)
		Chile	2 (3)
		Asia	3 (5)
	**Study objective (n=60)**		
		Process evaluation only	27 (45)
		Process and effects evaluation in one article	13 (22)
		Effects evaluation only	10 (17)
		Protocol study only	10 (17)
	**Measure for evaluation^b^ (n=60)**		
		Process evaluation (quantitative questionnaire)	21 (35)
		Process evaluation (qualitative measure)	21 (35)
		Effectiveness evaluation (quantitative questionnaire)	19 (32)
		Effectiveness evaluation (qualitative measure)	3 (5)
**Characteristics of interventions**			
	**Target population^b^ (n=37)**		
		General	19 (51)
		Specific	18 (49)
		Sexual orientation	8 (22)
		Ethnic minorities	4 (11)
		Others	7 (19)
	**Sex (n=37)**		
		Males and females	24 (65)
		Males only	8 (22)
		Females only	5 (14)
	**Age group (years; n=37)**		
		10 to 17	2 (5)
		10 to 24	13 (35)
		10 to >24	8 (22)
		18 to 24	4 (11)
		18 to >24	8 (22)
		Age not specified but considered as “students” or “youth”	2 (5)
	**Recruitment^b^ (n=34; NI^a^=3)**		
		Social networking sites	12 (35)
		Internet	11 (32)
		Secondary schools	9 (26)
		Community or youth organizations	8 (24)
		Clinics	7 (21)
		Universities	5 (15)
		Email	4 (12)
		Peers and word of mouth	3 (9)
		Phone	2 (6)
		Registers	1 (3)
		Smartphone apps	1 (3)
		Health educators	1 (3)
	**Incentives^b^ (n=23; NI^a^=14)**		
		Yes	21 (91)
		Direct remuneration	12 (52)
		Gift card	10 (43)
		Book or movie voucher	1 (4)
		Points for lot	1 (4)
		Raffle for remuneration	1 (4)
		No	2 (8)
	**Theme (n=37)**		
		Sexual health promotion	19 (51)
		HIV/sexually transmitted infection prevention specifically	12 (32)
		Sexual violence prevention	3 (8)
		Hepatitis B virus and hepatitis C virus testing promotion	1 (3)
		Improve HIV care linkage	1 (3)
		Observe peer influence in sexual situations only	1 (3)

^a^NI: no information in the article.

^b^For a given article (N=60) or an intervention (N=37), several entries are possible. Totals do not always equal 100%.

**Table 2:** Number of publications over time.

Year	Studies published, n
2006	1
2009	1
2010	3
2011	2
2012	8
2013	10
2014	4
2015	5
2016	9
2017	10
2018	6
2019 (January)	1

Descriptive data on the intervention types, online supports, and features are shown in Table 3 (for a description of each intervention, see Multimedia Appendix 4). Concerning intervention types, 41% (n=15/37) involve a dissemination of information with participatory components (game, quizzes, discussions). The medium used is a website in 54% (n=20/37) of cases, followed by online social networks (35%, n=13/37), with Facebook used in 22% (n=8/37) of cases. Furthermore, 14% (n=5/37) use several different online supports for the implementation of the intervention. To protect the identity of participants, 49% (n=18/37) of the interventions provide anonymity. Of these, 72% (n=13/18) allow participants to use personal identifiers, and 67% (n=12/18) use private websites. The interventions based on social networking sites do not mention anonymity because this is not possible on such sites. However, on Facebook, one (n=1) intervention used a secret group for greater confidentiality, another (n=1) used a private SMS text messaging system, and another (n=1) used a private page that only registered participants can access. Concerning participatory features, 68% allow interaction, either between peers (62%, n=23/37) or with a professional (43%, n=16/37). This interaction is mainly through online social networks (22%, n=8/37) and discussion forums (19%, n=7/37). Overall, 5% (n=2/37) use multiple supports for interaction. Involvement in a game-type activity was possible in 27% (n=10/37) of cases. Videos were broadcast in 54% (n=20/37) of cases. Finally, 43% (n=16/37) of the interventions were constructed from a theoretical model, with 19% (n=7/37) using the Information-Motivation-Behavioral Skills model.

**Table 3:** Intervention type, online support, and features description (N=37).

Variables			Studies, n (%)
**Intervention type**			
	Information dissemination with participatory components (games, quizzes, discussions)		15 (41)
	Online community/discussion only		11 (30)
	Participation in activities only (including games)		6 (16)
	Participatory educational session only		3 (8)
	Personalized assistance		2 (5)
**Online support for implementation^a^**			
	Website		20 (54)
	Social networking sites		13 (35)
	Online game only		5 (14)
	Apps		4 (11)
**Social networking sites used^a^**			
	Facebook		8 (22)
	YouTube		3 (8)
	MySpace		2 (5)
	Twitter		1 (3)
	Flickr		1 (3)
	Tumblr		1 (3)
	Instagram		1 (3)
	WeChat		1 (3)
	Not specified		1 (3)
**Participatory features (1) - interactive part^a^**			25 (68)
	Interaction between peers and with professionals		14 (38)
	Interaction between peers only		9 (24)
	Interaction with professionals only		2 (5)
	Peer leaders formation and implication		5 (14)
	Section to ask a professional		5 (14)
	**Support for interaction (peers and professionals)^a^**		
		Social networking sites	8 (22)
		Forum discussion	7 (19)
		Blog	3 (8)
		On website without more information	3 (8)
		Chat	2 (5)
		In the online game	2 (5)
		Video comment section	1 (3)
		On application	1 (3)
		“Ask the expert” section	1 (3)
**Participatory features (2) - involvement in an activity^a^**			16 (43)
	Online video game system		10 (27)
	Interactive quiz		4 (11)
	Personal goals		2 (5)
**Other features (3) - receipt of information^a^**			
	Video system		20 (54)
	Transmission or link of existing websites		4 (11)
**Theory model used for intervention conception^a^**			
	No		21 (57)
	**Yes**		16 (43)
		Information-Motivation-Behavioral Skills Model	7 (19)
		Social Identity Theory	2 (5)
		Social Cognitive Theory	2 (5)
		Social Learning Theory	1 (3)
		Others	9 (24)
		Two or more theories used	5 (14)
**Community-based participatory research**			
	Yes		21 (57)
	Unspecified		16 (43)

^a^An intervention can use several theories or several supports and contain different functionalities. Totals are not always equal to 100%.

The five most commonly used behavior change techniques are as follows (Multimedia Appendix 5). First, 78% (n=29/37) of interventions introduce or define an environmental or social stimulus to encourage or guide behavior. Second, 78% (n=29/37) provide information on the health consequences of performing the behavior. Third, 73% (n=27/37) present information from a credible source in favor of or against the behavior. Fourth, 70% (n=26/37) organize and provide some form of social support within the intervention. Fifth, 65% (n=24/37) provide information on what others think about the behavior. No intervention provides punitive measures or remuneration for the conduct of the behavior sought.

Of the 37 interventions, 57% (n=21/37) indicate that they called on young people for community-based participatory research (collective construction). This takes various forms: 38% (n=14) of the interventions conducted focus groups to discuss the proposed intervention, 27% (n=10) directly included youth in the development of content, 8% (n=3) adapted their content based on feedback from young people in pretest studies, 5% (n=2) involved youth in the evaluation, and 3% (n=1) formed a youth advisory committee.

Data on the design and evaluation methods are available in Table 4. For a description of the methods of each intervention, see Multimedia Appendix 6. In total, 43% (n=16/37) were evaluated according to a randomized controlled trial (RCT) design. Overall, 22% (n=8/37) provided a follow-up between 1 and 2 years, while the remainder reported a follow-up shorter than 1 year (59%, n=22/37) or did not specify a follow-up time (19%, n=7/37). For process evaluation, 35% (n=13/37) did an acceptability study, 30% (n=11/37) did an attractiveness study, and 27% (n=10/37) assessed feasibility. Regarding effectiveness, 46% (n=17/37) of the interventions were subject to an outcome evaluation and 32% (n=12/37) had a planned outcome evaluation. Among the outcomes evaluated (conducted or planned evaluation), sexual behaviors were the most evaluated (38%, n=14/37), followed by condom use (29%, n=11/37) and sexual health knowledge (22%, n=8/37).

**Table 4:** Intervention design and evaluation methodology (N=37).

Study information			Studies, n (%)
**Design study**			
	**Randomized controlled trial (RCT)**		16 (43)
		Control group (NI=2)^a,b^	15 (41)
		Information-only control website^b^	4 (11)
	Before-after study (no RCT)		7 (19)
	Cross-sectional study		3 (8)
	Other design		8 (22)
	Unspecified		3 (8)
**Follow-up**			
	No follow-up		3 (8)
	0.5-2 months		3 (8)
	3-5 months		9 (24)
	6-11 months		7 (19)
	12-24 months		8 (22)
	Unspecified		7 (19)
**Process outcomes evaluated^c^**			
	Acceptability		13 (35)
	Attractiveness		11 (30)
	Feasibility		10 (27)
	Satisfaction		3 (8)
	Implementation		3 (8)
**Outcomes evaluation conducted^c^**			17 (46)
	Behaviors		10 (27)
	Condom use, condom use intention, self-efficacy toward condom use, and attitude toward condom use		9 (24)
	Attitudes		4 (11)
	Communication		3 (8)
	Knowledge		3 (8)
	Behavioral skills		2 (5)
	Self-efficacy		2 (5)
	Contraception use		1 (3)
	History of sexually transmitted infections		1 (3)
	HIV stigma		1 (3)
	HIV test history (date and result of the last test)		1 (3)
	Incidence of sexually transmitted infections		1 (3)
	Intentions related to risky sexual activity		1 (3)
	Internalized homophobia		1 (3)
	Intimate partner violence		1 (3)
	Motivation		1 (3)
	Pubertal development		1 (3)
	Sexual abstinence		1 (3)
	Waiting before having sex		1 (3)
	Other outcomes evaluated only once		17 (46)
**Outcomes evaluation planned^c^**			12 (32)
	Knowledge		5 (14)
	Behaviors		4 (11)
	Condom use		2 (5)
	Intentions		2 (5)
	Self-efficacy		2 (5)
	Occurrence of pregnancy		1 (3)
	Occurrence of sexually transmitted infections		1 (3)
	Self-reported pregnancy		1 (3)
	Self-reported sexually transmitted infections		1 (3)
	Fertility distress		1 (3)
	Repeat HIV/sexually transmitted infection screening		1 (3)
	Number of tests for *Chlamydia trachomatis*		1 (3)
	HBsAg and anti–hepatitis C virus IgG test uptake		1 (3)
	HIV-related care engagement		1 (3)
	Motivation		1 (3)
	Number of partners		1 (3)
	Sexual communication self-efficacy		1 (3)
	Use of safety strategies		1 (3)
	Viral suppression		1 (3)
	Other outcomes planned for evaluation only once		7 (19)
**Unspecified outcomes evaluation**			8 (22)

^a^NI: no information in the article.

^b^Since a control group can also be a group receiving an informational website only, the total exceeds the number of RCTs.

^c^An intervention can evaluate several outcomes or process components. Totals are not always equal to 100%.

## Discussion

### Principal Results

Our review identified 37 different interventions, which were the subjects of 60 articles. The number of online participatory interventions for the promotion of young people's sexual health has increased significantly over the past 5 years, especially in the United States. Three key points drew our attention: (1) Several different online supports are used by interventions and we would recommend adapting these to young people's preferences; (2) Online peer interaction is the participatory element most often used in interventions and is a promising health promotion approach; (3) In view of the limited number of effectiveness evaluations, it is necessary to define a conceptual model of interventions to enable comprehensive and rigorous evaluation and to understand the effect of peer interaction and participatory components.

### How to Adapt to the Favorite Media of Young People?

Concerning the online support used, interventions are mainly first developed on websites. The second most popular medium is social networks, with Facebook dominating, as already shown in a previous review of social networking sites [18].

Surprisingly, young people's favorite social networks [27] are rarely used. Only one intervention was on Instagram [28], three were on YouTube, and none were on Snapchat. However, these three media have been described as the new preferred ones of youth, whereas the popularity of Facebook is declining [27]. The future challenge for researchers will be to develop interventions that can evolve with young people’s preferences, keeping up with rapid generational changes. In our review, few interventions use more than one online medium. One option would be to use a multichannel approach for interventions. Such an approach already exists to some extent in the American intervention “weCare,” which allows young people to choose how they connect with educators, with three possible contact modalities: Facebook Messenger, SMS text messaging, and app-based instant messages [29].

Our findings also highlight the need to design interventions adapted to the uses, languages, interests, and realities of young people, particularly through interactive and playful components. One way to remain close to the interests of young people is to integrate promising new media in interventions, such as videos and games. It is also possible to allow users to insert their own content or to customize websites. Integrating attractive components that are correctly implemented will ensure better group retention. To know what is preferred by young people, it is therefore necessary to have measures of attractiveness. This review has cited different measures: online media usage, process data (number of visits, time spent, and interaction rate), technical recommendations, content adapted to the target audience (specificity and age), satisfaction, points of view, and involvement of participants (especially sexual minorities).

Web-based interventions also raise the challenges of security, privacy, and anonymity. For example, the lower use of social networking sites for research compared to websites may also be due to the fact that the ownership of the data from youth participation belongs to these media. This data would be less easy to protect in terms of security, confidentiality, and privacy, especially against cyberstalking, requiring moderation at all times. In the studies reviewed here, authors provided little information on how they protected participants' data. On social networking sites, some researchers use closed groups to control the exchange of participants' data. Others host the data through a secure external website. Technical partners, such as social networking sites, are bound by specific laws and contractual data protection clauses, and there is a clear regulatory framework for many countries [30]. As noted by some authors [11,31,32], ethical and data security frameworks need to be strengthened. For example, the importance of blocking public access to online interventions and developing powerful security features is underlined [33]. Concerning anonymity, protection of the identity of participants is possible mainly on private websites, which is especially important in the context of sexual health, where the internet is used to avoid embarrassment and overcome privacy issues [34].

### How to Implement Peer Dynamics in Interventions?

All media can be used to disseminate information among young people, either top-down (from an educator to a young person) or cross-functionally (between peers). The interest of the 37 interventions assessed here rests on their participatory activities, of which peer interaction is the most frequent component.

Peer exchanges were described in different ways: counselling, experience-sharing, community involvement, personal stories, self-help, and peer support. Peers were considered not only as participants, but also as peer educators (opinion leaders) previously trained by professionals [35-37]. In one study, the potential for sharing and comparing real experiences was supported [38], with an expressed need for sharing experiences among peers. Participants also expressed the desire for social interaction online with other young people [39].

More personalized approaches better target the concerns of each individual, as seen in the Media Aware [40] and Queer Sex Ed [41] interventions (individuals’ goals). Participants could also disseminate their own content, as seen in the HealthMpowerment intervention [42-44]. Peer dynamics also occur when young people are directly involved in the community-based participatory research process, especially in sexuality education programs [10]. This process can validate the role of community members and academics as equitable partners [45]. In our review, we determined that this process is widely used at the design stage. Peer interaction is thus enabled by most interventions and is described as strengthening an intervention’s capacity to change behaviors, even if professionals are involved. The dynamics between peers, and the feeling of being “between young people,” are seen as potentialities. Surprisingly, the term “peer education” is not a term used in the reviewed articles. “Peer education” is actually an exchange of experiences and information between peers in “real life,” integrating the notion of “shared education” [46], and is thus well suited to these interventions. One intervention did use the term “peer-led” [35]. Peer dynamics are little conceptualized by the authors, and a model for designing and evaluating interventions is lacking.

### How to Evaluate Interventions?

The objective of interventions is to change sexual health outcomes positively. For the moment, although experimental plans are defined, publications focus more on intervention processes than effectiveness in terms of health outcomes. This probably reflects the need to identify implementation problems beforehand, as a lack of effect may reflect a failure in implementation rather than the ineffectiveness of the intervention [26]. Implementing an intervention correctly will ensure better group retention. To evaluate effectiveness, the randomized controlled trial remains the most widely used or planned design. It does not preclude assessing the effect of an intervention on a range of outcome measures [47].

In interventions dealing with evaluation, behaviors were most often the main outcome, followed by knowledge, self-efficacy, and attitudes. A majority of follow-up interventions lasted less than 1 year. Nonetheless, it would be interesting to have a long-term follow-up to determine whether short-term changes persist [21]. Behavior measures are based on self-reported data, and many authors have highlighted the issue of social desirability bias as a limitation [36,40,41,48-51].

Our review found few plans to observe a robust indicator, such as STI incidence [52], HIV-related care engagement and viral suppression [29], or pregnancy [53,54]. These indicators can measure the real impact of an intervention on sexual health. Nevertheless, this requires a large sample size in order to have sufficient power to detect the effects of the intervention, especially when the expected outcomes have a low baseline rate of incidence (eg, HIV incidence), unless these studies are conducted on high-risk groups.

In this context of complex intervention, mechanisms of action should be identified and interventions should rely on a theoretical, conceptual, and operational model. This will enable all the participatory, social, and collective variables involved in the process to be measured and validated. Based on a literature review, Borek and Abraham developed a conceptual model of mechanisms of change in small groups [55]. For peer interventions, Simoni et al [56] argue for a strong theoretical framework to support behavior promotion, link to outcomes, and justify peer inclusion. In addition, strategies combining several theories and concepts may have a greater effect [57], as seen in the TeensTalkHealth intervention [58], which used the IMB model [7] combined with communication theory [59]. Several interactive processes (group development, group dynamics, social change) have been highlighted and could be used for the constitution and animation of social groups [55]. Finally, applying a comprehensive model of internet-based peer education (or peer-led behavior change) for sexual health is a promising approach, as long as a proliferation of concept and theoretical models does not occur. Rigorous methods, such as the 5 steps of the Intervention Mapping protocol, can contribute to the development of more effective behavior change interventions and methods of evaluation, assessing all stages of adoption, implementation, and sustainability of the intervention [60,61].

### Limitations

Our review was conducted with a cross-validation methodology based on two search tools (PubMed and Aurore), but we cannot rule out that some interventions escaped our research. Participatory or interactive interventions may exist but may not be evaluated and published (for example, the website Sex, Etc [62]). Finally, wide variations in interventions made it inappropriate to synthesize the results using a meta-analysis.

### Conclusions

This review describes existing interventions in participatory sexuality education for young people on the internet. It aims to provide guidance for interventions that meet the expectations of national and international strategies on youth sexuality education. Identified interventions are deployed on many internet media and have shown their feasibility, practical interest, and attractiveness. However, they are still in the early stages of design and evaluation, particularly as regards the effect of peer interaction, and do not always adhere to existing theoretical models. We recommend building a conceptual, theoretical, and evaluation model for community-based interventions involving peer interaction and participation in activities, providing the necessary operational and evaluative tools. Interventions must be designed with regard to media multiplicity, youth populations (orientations, gender identities), and a holistic sexual health approach. To improve these interventions, we recommend having a more participatory approach, involving young people in the whole process, including the design phase.

## Appendix

Multimedia Appendix 1 Checklist items pertaining to the content of a systematic review and meta-analysis.

Multimedia Appendix 2 Research strategies used for PubMed research and Aurore complementary research.

Multimedia Appendix 3 All the studies included in the systematic review.

Multimedia Appendix 4 Description of the interventions included and their participatory components.

Multimedia Appendix 5 Coding of Michie's taxonomy on Behaviour Change Techniques.

Multimedia Appendix 6 Description of intervention studies, designs, and evaluation methods.

## Abbreviations

AYA: adolescents and young adults
BCT: behavior change techniques
IMB: Information-Motivation-Behavioral Skills
INED: Institut National d’Études Démographiques
RCT: randomized controlled trial
STI: sexually transmitted infection
WHO: World Health Organization
