# Supplementary material for: Participatory Interventions for Sexual Health Promotion for Adolescents and Young Adults on the Internet: Systematic Review
Source: J Med Internet Res. 2020 Jul 31;22(7):e15378. doi: 10.2196/15378 (PMC7428916; doi:10.2196/15378)
Supplement: Multimedia Appendix 2 [file jmir_v22i7e15378_app2.docx]

Multimedia Appendix 2: Search strategies

**PubMed search strategy**

("adolescents"[MeSH Terms] OR "young adults"[MeSH Terms] OR "youth"[Text Word] OR "young"[Text Word] OR "AYA"[Text Word]) AND ("reproductive health"[MeSH Terms] OR "sexual behavior"[MeSH Terms] OR "sexual health"[Text Word] OR "contraception"[Text Word] OR "sexuality"[Text Word] OR "Intimate Partner Violence"[MeSH Terms] OR "Sex Offenses"[MeSH Terms] OR "Domestic Violence"[MeSH Terms] OR "Child Abuse, Sexual"[MeSH Terms] OR "Sexual Harassment"[MeSH Terms] OR "sexual well-being"[Text Word] OR "sexual"[Text Word] OR "reproductive health"[Text Word] OR "Sexuality"[MeSH Terms] OR "Abortion, Induced"[MeSH Terms] OR "Contraception"[MeSH Terms] OR "Sexually Transmitted Diseases"[MeSH Terms] OR "pregnancy"[Text Word] OR "unwanted pregnancies"[Text Word] OR "unwanted pregnancy"[Text Word] OR "sexual coercion"[Text Word] OR "Sexology"[MeSH Terms] OR "Sexual Dysfunctions, Psychological"[MeSH Terms] OR "Contraception Behavior"[MeSH Terms] OR "Circumcision, Female"[MeSH Terms] OR "Circumcision, Male"[MeSH Terms] OR "genital mutilation"[Text Word] OR "Rape"[MeSH Terms] OR (("reproductive health"[MeSH Terms] OR "sexual behavior"[MeSH Terms] OR "sexual health"[Text Word] OR "contraception"[Text Word] OR "sexuality"[Text Word] OR "Intimate Partner Violence"[MeSH Terms] OR "Sex Offenses"[MeSH Terms] OR "Domestic Violence"[MeSH Terms] OR "Child Abuse, Sexual"[MeSH Terms] OR "Sexual Harassment"[MeSH Terms] OR "sexual well-being"[Text Word] OR "sexual"[Text Word] OR "reproductive health"[Text Word] OR "Sexuality"[MeSH Terms] OR "Abortion, Induced"[MeSH Terms] OR "Contraception"[MeSH Terms] OR "Sexually Transmitted Diseases"[MeSH Terms] OR "pregnancy"[Text Word] OR "unwanted pregnancies"[Text Word] OR "unwanted pregnancy"[Text Word] OR "sexual coercion"[Text Word] OR "Sexology"[MeSH Terms] OR "Sexual Dysfunctions, Psychological"[MeSH Terms] OR "Contraception Behavior"[MeSH Terms] OR "Circumcision, Female"[MeSH Terms] OR "Circumcision, Male"[MeSH Terms] OR "genital mutilation"[Text Word] OR "Rape"[MeSH Terms]) AND ("Self Concept"[MeSH Terms] OR "Interpersonal Relations"[MeSH Terms] OR "Social Change"[MeSH Terms] "Social Norms"[MeSH Terms] OR "Social Marginalization"[MeSH Terms] OR "Social Isolation"[MeSH Terms] OR "Social Conditions"[MeSH Terms] OR "Social Environment"[MeSH Terms] OR "social transformations"[Text Word]))) AND ("internet"[MeSH Terms] OR "online"[Text Word] OR "web-based" [Text Word] OR "internet"[Text Word] OR "website"[Text Word]) AND ("community participation"[MeSH Terms] OR "social participation"[MeSH Terms] OR "community"[Text Word] OR "participatory"[Text Word] OR "interaction"[Text Word] OR "health information exchange"[MeSH Terms] OR "Peer Group"[MeSH Terms] OR "Group Structure"[MeSH Terms] OR "Sensitivity Training Groups"[MeSH Terms] OR "Group Processes"[MeSH Terms] OR "peer sexual education"[Text Word] OR "peer education"[Text Word] OR "empowerment"[Text Word] OR "information"[Text Word] OR "networking"[Text Word] OR "social network sites"[Text Word] OR "social network websites"[Text Word] OR "blog"[Text Word] OR "forum"[Text Word] OR "facebook"[Text Word] OR "twitter"[Text Word] OR "snapchat"[Text Word] OR "instagram"[Text Word] OR "Video Games"[MeSH Terms] OR "myspace"[Text Word] OR "social network"[Text Word] OR "peer-to-peer"[Text Word] OR "peer to peer"[Text Word])

**AURORE database of INED search strategy**

"AB ( adolescents or teenagers or young adults or teen or youth or student or adolescence or young or AYA ) AND AB ( (reproductive health OR sexual behavior OR sexual health OR contraception OR sexuality OR Intimate Partner Violence OR Sex Offenses OR Domestic Violence OR Child Abuse, Sexual OR Sexual Harassment OR sexual well-being OR sexual OR Abortion, Induced OR Contraception OR Sexually Transmitted Diseases OR pregnancy OR unwanted pregnancies OR unwanted pregnancy OR sexual coercion OR Sexology OR Sexual Dysfunctions, Psychological OR Contraception Behavior OR Circumcision, Female OR Circumcision, Male OR genital mutilation OR Rape OR ((reproductive health OR sexual behavior OR sexual health OR contraception OR sexuality OR Intimate Partner Violence OR Sex Offenses OR Domestic Violence OR Child Abuse, Sexual OR Sexual Harassment OR sexual well-being OR sexual OR Abortion, Induced OR Contraception OR Sexually Transmitted Diseases OR pregnancy OR unwanted pregnancies OR unwanted pregnancy OR sexual coercion OR Sexology OR Sexual Dysfunctions, Psychological OR Contraception Behavior OR Circumcision, Female OR Circumcision, Male OR genital mutilation OR Rape) AND (Self Concept OR Interpersonal Relations OR Social Change Social Norms OR Social Marginalization OR Social Isolation OR Social Conditions OR Social Environment OR social transformations)) ) AND AB ( internet OR online OR web-based OR website ) AND AB ( community participation OR social participation OR community OR participatory OR interaction OR health information exchange OR Peer Group OR Group Structure OR Sensitivity Training Groups OR Group Processes OR peer sexual education OR peer education OR empowerment OR information OR networking OR social network sites OR social network websites OR blog OR forum OR facebook OR twitter OR snapchat OR instagram OR Video Games OR myspace OR social network OR peer-to-peer OR peer to peer )

**AURORE research strategy for the following databases**

• OpenAIRE n=417

• ERICn=238

• ScienceDirect n=226

• ProjectMUSEn=106

• Directory of Open Access Journalsn=87

• FRANCIS Archiven=58

• SwePubn=27

• SciELOn=22

• British Library EThOSn=22

• JSTOR Journalsn=16

• RePEc n=9

• Emerald Insightn=9

• ICPSR Data Archive n=7

• SSOAR – Social Science Open Access Repositoryn=3

• Openedition.orgn=3

• Gale Virtual Reference Libraryn=2

• Erudit n=2

• Books at JSTOR n=1

• OAPEN Library n=1

Types of sources

• University Journals (470)

• Magazines (267)

• Reports (239)

• Books (132)

• Dissertations / Theses (79)
