# Supplementary material for: Participatory Interventions for Sexual Health Promotion for Adolescents and Young Adults on the Internet: Systematic Review
Source: J Med Internet Res. 2020 Jul 31;22(7):e15378. doi: 10.2196/15378 (PMC7428916; doi:10.2196/15378)
Supplement: Multimedia Appendix 3 [file jmir_v22i7e15378_app3.docx]

# Multimedia Appendix 3: Studies included by the systematic review

1. Markham CM, Shegog R, Leonard AD, Bui TC, Paul ME. +CLICK: harnessing web-based training to reduce secondary transmission among HIV-positive youth. AIDS Care. 2009 May;21(5):622-31. PMID: 19444671. DOI: 10.1080/09540120802385637.

2. Lou C, Zhao Q, Gao E-S, Shah IH. Can the Internet be used effectively to provide sex education to young people in China? J Adolesc Health. 2006 Nov;39(5):720-8. PMID: 17046509. DOI: 10.1016/j.jadohealth.2006.04.003

3. Sun WH, Wong CKH, Wong WCW. A Peer-Led, Social Media-Delivered, Safer Sex Intervention for Chinese College Students: Randomized Controlled Trial. J Med Internet Res. 2017 Aug 9;19(8):e284. PMID: 28793980. DOI: 10.2196/jmir.7403

4. Widman L, Choukas-Bradley S, Helms SW, Prinstein MJ. Adolescent Susceptibility to Peer Influence in Sexual Situations. J Adolesc Health. 2016 Mar;58(3):323-9. PMID: 26794431. DOI: 10.1016/j.jadohealth.2015.10.253.

5. Fitzpatrick T, Zhou K, Cheng Y, Chan P-L, Cui F, Tang W, et al. A crowdsourced intervention to promote hepatitis B and C testing among men who have sex with men in China: study protocol for a nationwide online randomized controlled trial. BMC Infect Dis. 2018 Sep 29;18(1):489. PMID: 30268114. DOI: 10.1186/s12879-018-3403-3.

6. Ybarra ML, DuBois LZ, Parsons JT, Prescott TL, Mustanski B. Online focus groups as an HIV prevention program for gay, bisexual, and queer adolescent males. AIDS Educ Prev. 2014 Dec;26(6):554-64. PMID: 25490735. DOI: 10.1521/aeap.2014.26.6.554.

7. Bull S, Nabembezi D, Birungi R, Kiwanuka J, Ybarra M. Cyber-Senga: Ugandan youth preferences for content in an internet-delivered comprehensive sexuality education programme. East Afr J Public Health. 2010 Mar;7(1):58-63. PMID: 21413574.

8. Ybarra ML, Biringi R, Prescott T, Bull SS. Usability and navigability of an HIV/AIDS internet intervention for adolescents in a resource-limited setting. Comput Inform Nurs. 2012 Nov;30(11):587-95; quiz 596-7. PMID: 22918136. DOI: 10.1097/NXN.0b013e318266cb0e.

9. Ybarra ML, Bull SS, Prescott TL, Birungi R. Acceptability and feasibility of CyberSenga: an Internet-based HIV-prevention program for adolescents in Mbarara, Uganda. AIDS Care. 2014 Apr;26(4):441-7. PMID: 24093828. DOI: 10.1080/09540121.2013.841837.

10. Ybarra ML, Bull SS, Prescott TL, Korchmaros JD, Bangsberg DR, Kiwanuka JP. Adolescent abstinence and unprotected sex in CyberSenga, an Internet-based HIV prevention program: randomized clinical trial of efficacy. PLoS One. 2013 Aug 14;8(8):e70083. PMID: 23967069. DOI: 10.1371/journal.pone.0070083.

11. Gold J, Pedrana AE, Stoove MA, Chang S, Howard S, Asselin J, et al. Developing Health Promotion Interventions on Social Networking Sites: Recommendations from The FaceSpace Project. J Med Internet Res. 2012 Feb 28;14(1):e30. PMID: 22374589. DOI: 10.2196/jmir.1875.

12. Nguyen P, Gold J, Pedrana A, Chang S, Howard S, Ilic O, et al. Sexual health promotion on social networking sites: a process evaluation of The FaceSpace Project. J Adolesc Health. 2013 Jul;53(1):98-104. PMID: 23583509. DOI: 10.1016/j.jadohealth.2013.02.007.

13. van Rosmalen-Nooijens KAWL, Prins JB, Vergeer M, Wong SHLF, Lagro-Janssen ALM. « Young people, adult worries »: RCT of an internet-based self-support method « Feel the ViBe » for children, adolescents and young adults exposed to family violence, a study protocol. BMC Public Health. 2013 Mar 15;13:226. PMID: 23497359. DOI: 10.1186/1471-2458-13-226.

14. Winterling J, Wiklander M, Obol CM, Lampic C, Eriksson LE, Pelters B, et al. Development of a Self-Help Web-Based Intervention Targeting Young Cancer Patients With Sexual Problems and Fertility Distress in Collaboration With Patient Research Partners. JMIR Res Protoc. 2016 Apr 12;5(2):e60. PMID: 27073057. DOI: 10.2196/resprot.5499.

15. Mak DB, Bastian L, Grace J, Aquilina H, Sweeting J. Evaluation of a sexual health and blood-borne virus health education website for youth. Health Promot J Austr. 2012 Dec;23(3):194-200. PMID: 23540319

16. Rice E, Tulbert E, Cederbaum J, Barman Adhikari A, Milburn NG. Mobilizing homeless youth for HIV prevention: a social network analysis of the acceptability of a face-to-face and online social networking intervention. Health Educ Res. 2012 Apr;27(2):226-36. PMID: 22247453. DOI: 10.1093/her/cyr113.

17. Hightow-Weidman LB, Pike E, Fowler B, Matthews DM, Kibe J, McCoy R, et al. HealthMpowerment.org: feasibility and acceptability of delivering an internet intervention to young Black men who have sex with men. AIDS Care. 2012;24(7):910-20. PMID: 22272759. DOI: 10.1080/09540121.2011.647677.

18. Hightow-Weidman LB, LeGrand S, Muessig KE, Simmons RA, Soni K, Choi SK, et al. A Randomized Trial of an Online Risk Reduction Intervention for Young Black MSM. AIDS Behav. 2019 May;23(5):1166-1177. PMID: 30269231. DOI: 10.1007/s10461-018-2289-9

19. Bauermeister JA, Muessig KE, LeGrand S, Flores DD, Choi SK, Dong W, et al. HIV and Sexuality Stigma Reduction Through Engagement in Online Forums: Results from the HealthMPowerment Intervention. AIDS Behav. 2019 Mar;23(3):742-752. PMID: 30121727. DOI: 10.1007/s10461-018-2256-5.

20. Hightow-Weidman LB, Fowler B, Kibe J, McCoy R, Pike E, Calabria M, et al. HealthMpowerment.org: development of a theory-based HIV/STI website for young black MSM. AIDS Educ Prev. 2011 Feb;23(1):1-12. PMID: 21341956. DOI: 10.1521/aeap.2011.23.1.1.

21. Barry MC, Threats M, Blackburn NA, LeGrand S, Dong W, Pulley DV, Sallabank G, Harper GW, Hightow-Weidman LB, Bauermeister JA, Muessig KE. « Stay strong! keep ya head up! move on! it gets better!!!! »: resilience processes in the healthMpowerment online intervention of young black gay, bisexual and other men who have sex with men. AIDS Care. 2018 Aug;30(sup5):S27-S38. PMID: 30632775. DOI: 10.1080/09540121.2018.1510106.

22. Williams M, Bowen A, Ei S. An evaluation of the experiences of rural MSM who accessed an online HIV/AIDS health promotion intervention. Health Promot Pract. 2010 Jul;11(4):474-82. PMID: 19116419. DOI: 10.1177/1524839908324783.

23. O’Donnell NH, Willoughby JF. Photo-sharing social media for eHealth: analysing perceived message effectiveness of sexual health information on Instagram. J Vis Commun Med. 2017 Oct;40(4):149-159. PMID: 29022412. DOI: 10.1080/17453054.2017.1384995.

24. Ko N-Y, Hsieh C-H, Wang M-C, Lee C, Chen C-L, Chung A-C, et al. Effects of Internet popular opinion leaders (iPOL) among Internet-using men who have sex with men. J Med Internet Res. 2013 Feb 25;15(2):e40. PMID: 23439583. DOI: 10.2196/jmir.2264.

25. Villegas N, Santisteban D, Cianelli R, Ferrer L, Ambrosia T, Peragallo N, et al. Pilot testing an internet-based STI and HIV prevention intervention with Chilean women. J Nurs Scholarsh. 2015 Mar;47(2):106-16. PMID: 25410132. DOI: 10.1111/jnu.12114.

26. Villegas N, Santisteban D, Cianelli R, Ferrer L, Ambrosia T, Peragallo N, et al. The development, feasibility and acceptability of an Internet-based STI-HIV prevention intervention for young Chilean women. Int Nurs Rev. 2014 Mar;61(1):55-63. PMID: 24512261. DOI: 10.1111/inr.12080.

27. Bull SS, Levine DK, Black SR, Schmiege SJ, Santelli J. Research article: Social Media–Delivered Sexual Health Intervention. A Cluster Randomized Controlled Trial. Am J Prev Med. 2012 Nov;43(5):467-74. PMID: 23079168. DOI: 10.1016/j.amepre.2012.07.022.

28. Bull SS, Breslin LT, Wright EE, Black SR, Levine D, Santelli JS. Case study: An ethics case study of HIV prevention research on Facebook: the Just/Us study. J Pediatr Psychol. 2011 Nov-Dec;36(10):1082-92. PMID: 21292724. DOI: 10.1093/jpepsy/jsq126.

29. Bull SS, Levine D, Schmiege S, Santelli J. Recruitment and retention of youth for research using social media: Experiences from the Just/Us study. Vulnerable Child Youth Stud. 1 juin 2013;8(2):171‑81.

30. Greene GJ, Madkins K, Andrews K, Dispenza J, Mustanski B. Implementation and Evaluation of the Keep It Up! Online HIV Prevention Intervention in a Community-Based Setting. AIDS Educ Prev. 2016 Jun;28(3):231-45. PMID: 27244191. DOI: 10.1521/aeap.2016.28.3.231.

31. Mustanski B, Garofalo R, Monahan C, Gratzer B, Andrews R. Feasibility, acceptability, and preliminary efficacy of an online HIV prevention program for diverse young men who have sex with men: the keep it up! intervention. AIDS Behav. 2013 Nov;17(9):2999-3012. PMID: 23673793. DOI: 10.1007/s10461-013-0507-z.

32. Mustanski B, Parsons JT, Sullivan PS, Madkins K, Rosenberg E, Swann G. Biomedical and Behavioral Outcomes of Keep It Up!: An eHealth HIV Prevention Program RCT. Am J Prev Med. 2018 Aug;55(2):151-158. PMID: 29937115. DOI: 10.1016/j.amepre.2018.04.026.

33. Motley DN, Hammond S, Mustanski B. Strategies Chosen by YMSM During Goal Setting to Reduce Risk for HIV and Other Sexually Transmitted Infections: Results From the Keep It Up! 2.0 Prevention Trial. AIDS Educ Prev. 2017 Feb;29(1):1-13. PMID: 28195780. DOI: 10.1521/aeap.2017.29.1.1.

34. Mustanski B, Madkins K, Greene GJ, Parsons JT, Johnson BA, Sullivan P, et al. Internet-Based HIV Prevention With At-Home Sexually Transmitted Infection Testing for Young Men Having Sex With Men: Study Protocol of a Randomized Controlled Trial of Keep It Up! 2.0. JMIR Res Protoc. 2017 Jan 7;6(1):e1. PMID: 28062389. DOI: 10.2196/resprot.5740.

35. Gilliam M, Jagoda P, Jaworski E, Hebert LE, Lyman P, Wilson MC. « Because if We Don’t Talk about It, How Are We Going to Prevent It? »: « Lucidity, » a Narrative-Based Digital Game about Sexual Violence. Sex Educ Sex Soc Learn. 1 janv 2016;16(4):391‑404.

36. Scull TM, Kupersmidt JB, Malik CV, Keefe EM. Examining the Efficacy of an mHealth Media Literacy Education Program for Sexual Health Promotion in Older Adolescents Attending Community College. J Am Coll Health. 2018 Apr;66(3):165-177. PMID: 29068772. DOI: 10.1080/07448481.2017.1393822.

37. Campo S, Askelson NM, Spies EL, Losch M. Caution, the Use of Humor May Lead to Confusion: Evaluation of a Video Podcast of the Midwest Teen Sex Show. Am J Sex Educ. 2010 jan 1;5(3):201‑16.

38. Chen E, Barrington C. « You Can Do It Anywhere »: Student and Teacher Perceptions of an Online Sexuality Education Intervention. Am J Sex Educ. 2017 jan 1;12(2):105‑19.

39. Payton FC, Kvasny L. Online HIV awareness and technology affordance benefits for black female collegians - maybe not: the case of stigma. J Am Med Inform Assoc. 2016 Nov;23(6):1121-1126. PMID: 27094988. DOI: 10.1093/jamia/ocw017.

40. Glass N, Clough A, Case J, Hanson G, Barnes-Hoyt J, Waterbury A, et al. A safety app to respond to dating violence for college women and their friends: the MyPlan study randomized controlled trial protocol. BMC Public Health. 2015 Sep 8;15:871. PMID: 26350482. DOI: 10.1186/s12889-015-2191-6.

41. Alhusen J, Bloom T, Clough A, Glass N. Development of the MyPlan Safety Decision App with Friends of College Women in Abusive Dating Relationships. J Technol Hum Serv. 2015 July ;33(3):263‑82.

42. Lindsay M, Messing JT, Thaller J, Baldwin A, Clough A, Bloom T, et al. Survivor Feedback on a Safety Decision Aid Smartphone Application for College-Age Women in Abusive Relationships. J Technol Hum Serv. 2013oct; 31(4):368‑88.

43. Draper JL. SaVE Our Campus: Analyzing the Effectiveness of an Online Sexual Violence Program [Internet]. ProQuest LLC; 2017. Avalable on: https://rucore.libraries.rutgers.edu/rutgers-lib/52046/PDF/1/play/

44. Oliveira RNG de, Gessner R, Souza V de, Fonseca RMGS da. Limites e possibilidades de um jogo online para a construção de conhecimento de adolescentes sobre a sexualidade / Limits and possibilities of an online game for building adolescents’ knowledge of sexuality. Cien Saude Colet. 2016 Aug;21(8):2383-92. PMID: 27557012. DOI: 10.1590/1413-81232015218.04572016.

45. Souza V de, Gazzinelli MF, Soares AN, Fernandes MM, Oliveira RNG de, Fonseca RMGS da. The game as strategy for approach to sexuality with adolescents: theoretical-methodological reﬂections. Rev Bras Enferm. 2017 Apr;70(2):376-383. PMID: 28403303. DOI: 10.1590/0034-7167-2016-0043.

46. Fiellin LE, Kyriakides TC, Hieftje KD, Pendergrass TM, Duncan LR, Dziura JD, et al. The design and implementation of a randomized controlled trial of a risk reduction and human immunodeficiency virus prevention videogame intervention in minority adolescents: PlayForward: Elm City Stories. Clin Trials. 2016 Aug;13(4):400-8. PMID: 27013483. DOI: 10.1177/1740774516637871.

47. Hieftje K, Fiellin LE, Pendergrass T, Duncan LR. Development of an HIV Prevention Videogame Intervention: Lessons Learned. Int J Serious Games. 2016;3:83–90.

48. Duncan LR, Hieftje KD, Culyba S, Fiellin LE. Game playbooks: tools to guide multidisciplinary teams in developing videogame-based behavior change interventions. Transl Behav Med. 2014 Mar;4(1):108-16. PMID: 24653781. DOI: 10.1007/s13142-013-0246-8.

49. Mustanski B, Greene GJ, Ryan D, Whitton SW. Feasibility, acceptability, and initial efficacy of an online sexual health promotion program for LGBT youth: the Queer Sex Ed intervention. J Sex Res. 2015;52(2):220-30. PMID: 24588408. DOI: 10.1080/00224499.2013.867924.

50. Yeo TED, Chu TH. Sharing « Sex Secrets » on Facebook: A Content Analysis of Youth Peer Communication and Advice Exchange on Social Media about Sexual Health and Intimate Relations. J Health Commun. 2017 Sep;22(9):753-762. PMID: 28796578. DOI: 10.1080/10810730.2017.1347217.

51. Nicholas A, Murray E, Bailey JV, Stevenson F. The Sexunzipped trial: young people’s views of participating in an online randomized controlled trial. J Med Internet Res. 2013 Dec 12;15(12):e276. PMID: 24334198. DOI: 10.2196/jmir.2647

52. Bailey JV, Pavlou M, Copas A, McCarthy O, Carswell K, Rait G, et al. The Sexunzipped trial: optimizing the design of online randomized controlled trials. J Med Internet Res. 2013 Dec 11;15(12):e278. PMID: 24334216. DOI: 10.2196/jmir.2668.

53. Bailey J, Carswell K, Murray E, McCarthy O. Integrating psychological theory into the design of an online intervention for sexual health: the sexunzipped website. JMIR Res Protoc. 2012 Nov 19;1(2):e16. PMID: 23612122. DOI: 10.2196/resprot.2114.

54. McCarthy O, Carswell K, Murray E, Free C, Stevenson F, Bailey JV. What young people want from a sexual health website: design and development of Sexunzipped. J Med Internet Res. 2012 Oct 12;14(5):e127. PMID: 23060424. DOI: 10.2196/jmir.2116.

55. Danielson CK, McCauley JL, Jones A, Borkman AO, Miller S, Ruggiero KJ. Feasibility of Delivering Evidence-Based HIV/STI Prevention Programming to A Community Sample of African-American Teen Girls via the Internet. AIDS Educ Prev. 2013 Oct;25(5):394-404. PMID: 24059877. DOI: 10.1521/aeap.2013.25.5.394.

56. Nielsen A, De Costa A, Bågenholm A, Danielsson KG, Marrone G, Boman J, Salazar M, Diwan V. Trial protocol: a parallel group, individually randomized clinical trial to evaluate the effect of a mobile phone application to improve sexual health among youth in Stockholm County. BMC Public Health. 2018 Feb 5;18(1):216. PMID: 29402241. DOI: 10.1186/s12889-018-5110-9.

57. Mejia CM, Acland D, Buzdugan R, Grimball R, Natoli L, McGrath MR, et al. An Intervention Using Gamification to Increase Human Immunodeficiency Virus and Sexually Transmitted Infection Screening Among Young Men Who Have Sex With Men in California: Rationale and Design of Stick To It. JMIR Res Protoc. 2017 Jul 17;6(7):e140. PMID: 28716771. DOI: 10.2196/resprot.8064.

58. Brady SS, Sieving RE, Terveen LG, Rosser BRS, Kodet AJ, Rothberg VD. An Interactive Website to Reduce Sexual Risk Behavior: Process Evaluation of TeensTalkHealth. JMIR Res Protoc. 2015 Sep 2;4(3):e106. PMID: 26336157. DOI: 10.2196/resprot.3440.

59. Zhang Q, Huhn KJ, Tan A, Douglas RE, Li HG, Murti M, et al. « Testing is Healthy » TimePlay campaign: Evaluation of sexual health promotion gamification intervention targeting young adults. Can J Public Health. 2017 Apr 20;108(1):e85-e90. PMID: 28425904. DOI: 10.17269/cjph.108.5634.

60. Tanner AE, Mann L, Song E, Alonzo J, Schafer K, Arellano E, et al. weCARE: A Social Media-Based Intervention Designed to Increase HIV Care Linkage, Retention, and Health Outcomes for Racially and Ethnically Diverse Young MSM. AIDS Educ Prev. 2016 Jun;28(3):216-30. PMID: 27244190. DOI: 10.1521/aeap.2016.28.3.216.
