# Supplementary material for: Participatory Interventions for Sexual Health Promotion for Adolescents and Young Adults on the Internet: Systematic Review
Source: J Med Internet Res. 2020 Jul 31;22(7):e15378. doi: 10.2196/15378 (PMC7428916; doi:10.2196/15378)
Supplement: Multimedia Appendix 4 [file jmir_v22i7e15378_app4.docx]

### **Multimedia Appendix 4 : Intervention description**

| Intervention | Intervention objective | Target population | Intervention participation description | Support | Peers interact | Interact with pro | Terms of interactions |
| --- | --- | --- | --- | --- | --- | --- | --- |
| "+CLICK"(1) | Enhance sexual risk reduction skills | Young people with HIV | Animation, interactive activities, peer and expert video (activities adapted from "It's Your Game, Keep it Real"). | Web-based application | Not described | Not described | Not described |
| No name (2) | Educate in sexual health | General population | Intervention that offered sexual and reproductive health knowledge, service information, counseling and discussion. | Website | Yes | Yes | AYA discussions (forum with supervision) |
| No name (3) | Promote safer sex | General population | Intervention developed by trained peer educators. Secret group for peer-to-peer discussion. | Facebook | Yes | No | Peer-to-peer discussion (with peers leader) |
| No name (4) | Identify predictors of susceptibility to peer influence in sexual situations | Rural, low-income middle schools AYA | Participants seated at computer workstations, "logged in" to the chat room, and connected with 3 other computer-generated students. | Website (Chat room) | Yes | No | Others students preprogrammed (not real) |
| No name (5) | Promote HBV and HCV testing | Men who have sex with men | Crowdsourced intervention: two images and two one-minute videos to men delivered every other day (total of 8 days). | WeChat | Not described | Not described | Not described |
| No name (6) | Preventing HIV | Gay, bisexual, queer | Focus group online as intervention: questions posted every day; participants answer moderators' questions and discuss with other members. | Website (Online focus group) | Yes | Yes | Discussion forum |
| CyberSenga (7–10) | Reduction in HIV risk behaviors | General population | Comprehensive sexuality education program. Six modules (1 hour per module). Puzzle as a game. | Website | Not described | Not described | Not described |
| FaceSpace project (11,12) | Promote Sexual Health (to disseminate sexual health messages) | General population | Fictional characters with a page for each (status updates, photos). Videos of the characters, texts intended to encourage dialogue. Quizzes to encourage users to consult pages and to interact. | Facebook, YouTube, MySpace, Twitter, Flickr | Yes | Yes | Discussions and interactions on social networks sites |
| Feel The Vibe (13) | To offer peer support, information on family violence in broad sense (dating violence, sexual health) and information about healthcare services | Population with family violence | Self-help and peer support through a forum, generated chats and the ability to “ask the expert” function. News page. | Website | Yes | Yes | Forum discussion, chat and "ask the expert" |
| Fex-Can Project (14) | To alleviate sexual problems and fertility-related distress | Cancer patients | Self-help on sexuality and infertility. Educational and behavior change content, multimedia, interactive online activities, and partial feedback support (discussion forum, feedback from experts). | Website | Yes | Yes | Discussions, testimonials and answers to publications (forum) |
| Get the Facts (15) | Preventing STI | General population | Information. "True Life Story" competition promoted through schools in which students were invited to submit a personal story for publication on the website. | Website | Not described | Not described | Not described |
| Have You Heard (16) | Preventing HIV | Youth homeless | Peer leaders trained to engage face-to-face peers in the creation of digital media. Participants invited "friends" from their social networks to join group pages for discussions (condom, HIV testing). | MySpace, Facebook | Yes | Not described | Discussion on Myspace and Facebook groups |
| HealthMpowerment.org (17–21) | Preventing HIV | Young black men who have sex with men | Supportive community to share experiences and discuss stigma-related content. "Ask Dr. W" section to post anonymous questions to a doctor. Share, comment on multimedia content created or linked to the web. | Website, YouTube | Yes | Yes | Exchanges on forum with discussions created by the participants |
| HOPE (22) | Preventing HIV | Rural Men Who Have Sex With Men | Three modules, each consisting of two 20-minute sessions. Virtual interactions observed by the participant and possibility to ask to know more. | Website | No | Yes | Ask professional for more information |
| In Case You're Curious (ICYC) (23) | Promote Sexual Health | General population | Participants text, email, or directly message with their questions and then professionals post an anonymous version of the question and an answer on the social media page. | Instagram | Yes | Yes | Instagram comments |
| iPOL (24) | Preventing HIV (HIV testing, risky behaviors) | Men Who Have Sex With Men | Investigators have trained opinion leaders (iPOL) so they can act on the group page with clips, news, videos, personal accounts and discussions about risky behaviors | Facebook | Yes | No | Discussions around topics brought by iPOLs |
| I-STIPI (25,26) | Preventing HIV/STI/domestic violence and addiction | General population | Information on different topics. Quizzes, blog to discuss the content of the session. | Website | Yes | NR | Discussion blog |
| Just/Us (27–29) | Preventing HIV and promote Sexual Health | General population | Facebook page (8 topics per week). Youth facilitators make multiple updates each day in the form of video links, quizzes, and games as well as threaded discussions. | Facebook | Yes | Yes | Discussion on Facebook page |
| Keep It Up! (30–34) | Preventing HIV | Young cisgender men who have sex with men | Discussions of community involvement, scenarios on hooking-up online, communication skills. Information is presented in various formats like games, animation, and peers’ videos. | Website | Not described | Not described | / |
| Lucidity (35) | Promote reflection and communication about sexual violence and health topics | General population | Four mini-games: room-escape puzzle, point-and-click adventure, educational fill-in-the blank challenge, three-dimensional maze. Links to external websites. | Online game | Not described | Yes | / |
| Media Aware (36) | Promote Sexual Health | General population | Five-lesson comprehensive program. Interactive features: quizzes with feedback, videos of peers, popular media examples, and skills practice with real-time feedback, program personalization (personalized profile, personal goals). | Website | No | No | / |
| Midwest Teen Sex Show (MTSS) (37) | Promote sexual health (by humor in intervention) | General population | Online video show. Interactive features, online community with videos, groups, a forum, blog, and social networking pages. | Website, Social Networking pages | Yes | Not described | Online community with exchanges on blog and forums |
| MyHealthEd (38) | Promote sexual health (sexuality education) | General population | Chat simulator screenshot. To transform scripted role play activities to chat simulator by customizing role plays and allowing the chat simulator to tailor to students' responses. | Website | No | No | / |
| myHealthImpactNetwork (39) | Promote awareness of HIV prevention | Black female college students | Involvement of students in the design of the platform. Zones of exchange between peers possible on blog. | Website, Tumblr, Twitter, YouTube | Yes | No | Blogging areas |
| MyPlan (40–42) | Provides a personalized safety plan to prevent Intimate Partner Violence | Dating Violence survivors and their friends | Security decision app: the user enters information about: a) the health of the relationship; (b) security priorities; and (c) the severity of the violence / danger in the relationship. | Web-based smartphone application (App) | Not described | Not described | / |
| Not Anymore (43) | Engage students in training on sexual violence | General population | A 60-minute, customizable content online program that engages students in sexual violence training through the use of animated graphics, animations, survivor stories and spectator videos. | Website | Yes | Yes | Interactive Multimedia |
| Papo Reto (44,45) | Construction of knowledge in the field of sexuality | General population | Virtual city made up of Home, School, Internet, Parties and Street, gradually unlocked by the participants' scores. Problematic situations are presented to participant to discuss, propose and share way of thinking and acting. | Online game | Yes | Yes | Comments between players, Life situations, reactions to comments |
| PlayForward: Elm City Stories (46–48) | To decrease risk and prevent HIV infection | At-risk young minority adolescents | Interactive world where players "travel" through time using an avatar, facing challenges such as peer pressure to drink alcohol or engage in risky sexual behaviors. | Online game (tablet-based videogame) | Not described | No | / |
| Queer Sex Ed (49) | Promote Sexual Health | LGBT Youth | Five modules. An avatar (Ed) served as the moderator. Each module ended with a quiz about the presented materials. If a question was answered incorrectly, the correct answer was explained. Selection of Individualized Sexual Health Improvement Goals. | Website, Facebook | Not described | Not described | / |
| Sex Secrets (50) | Provide peer resource on sexual health and intimate relations | General population | "Sex secrets" confession page for exchanging tips and answers to questions. Several messages posted daily- page used in de-identified manner. | Facebook | Yes | No | Comments and peer counseling on the social network page |
| Sexunzipped (51–54) | Promote sexual well-being (sexual health) | General population | Each section contains a combination of interactive quizzes and decision-making activities (text-based information). Links to other topics on the site at the end of all activities and text-based pages. | Website | Yes | Yes | Quotations: peer-to-peer exchange of views |
| Sihle Web (55) | Preventing HIV / STI | African-American girls | Four sessions simulating the experience of live group participation by using an interactive, video-based design. Interactive activities to complete (with real-time feedback from their video peers, Health Educator, and Near Peer). | Website | Yes | Yes | Comments on the videos |
| Skyddslaget (56) | Improve sexual health and practices (condom use and C.trachomatis positivity) | General population | Information excerpts on safe sex and STI with interactive and engaging elements including weekly challenges (games, quizzes) and personal stories from peers | Web-based application | Yes | Not described | Personal stories related to sexuality peers |
| Stick to it! (57) | Preventing HIV / STI | Young Men Who Have Sex With Men | 3 components: (1) online enrollment; (2) web-based activities (quizzes and countdown "timer" to facilitate screening reminders); (3) in-person activities that occur at 2 sexual health clinics. | Online game on website | No | No | / |
| TeensTalkHealth (58) | Promote condom use and other health behaviors | General population | Interactive intervention: information, thumbnails, videos, articles and discussion forums. 60 tasks to complete. | Website | Yes | Yes | Discussions on forum with conversation catalysts |
| Testing is Healthy (game: TimePlay) (59) | 1) Reduce misperceptions around STIs, including HIV, 2) Raise awareness of where and how to get tested, 3) Increase testing for STI/HIV | General population | Campaign "Testing is Healthy" delivered through TimePlay, an interactive game released before Cineplex feature films. | Online game on mobile application | No | No | / |
| weCare (60) | To improve care linkage and retention and health outcomes | Men Who Have Sex With Men Afro American and Latino | weCare Health Educator communicate with each participant individually using theory-based messages specific to each participant's place on the Continuum | Facebook messenger, text messaging, and app-based instant messages | Yes | Yes | Exchanges between peers and the professional on closed group |

1. Markham CM, Shegog R, Leonard AD, Bui TC, Paul ME. +CLICK: harnessing web-based training to reduce secondary transmission among HIV-positive youth. AIDS Care. 2009 May;21(5):622-31. PMID: 19444671. DOI: 10.1080/09540120802385637.

2. Lou C, Zhao Q, Gao E-S, Shah IH. Can the Internet be used effectively to provide sex education to young people in China? J Adolesc Health. 2006 Nov;39(5):720-8. PMID: 17046509. DOI: 10.1016/j.jadohealth.2006.04.003

3. Sun WH, Wong CKH, Wong WCW. A Peer-Led, Social Media-Delivered, Safer Sex Intervention for Chinese College Students: Randomized Controlled Trial. J Med Internet Res. 2017 Aug 9;19(8):e284. PMID: 28793980. DOI: 10.2196/jmir.7403

4. Widman L, Choukas-Bradley S, Helms SW, Prinstein MJ. Adolescent Susceptibility to Peer Influence in Sexual Situations. J Adolesc Health. 2016 Mar;58(3):323-9. PMID: 26794431. DOI: 10.1016/j.jadohealth.2015.10.253.

5. Fitzpatrick T, Zhou K, Cheng Y, Chan P-L, Cui F, Tang W, et al. A crowdsourced intervention to promote hepatitis B and C testing among men who have sex with men in China: study protocol for a nationwide online randomized controlled trial. BMC Infect Dis. 2018 Sep 29;18(1):489. PMID: 30268114. DOI: 10.1186/s12879-018-3403-3.

6. Ybarra ML, DuBois LZ, Parsons JT, Prescott TL, Mustanski B. Online focus groups as an HIV prevention program for gay, bisexual, and queer adolescent males. AIDS Educ Prev. 2014 Dec;26(6):554-64. PMID: 25490735. DOI: 10.1521/aeap.2014.26.6.554.

7. Bull S, Nabembezi D, Birungi R, Kiwanuka J, Ybarra M. Cyber-Senga: Ugandan youth preferences for content in an internet-delivered comprehensive sexuality education programme. East Afr J Public Health. 2010 Mar;7(1):58-63. PMID: 21413574.

8. Ybarra ML, Biringi R, Prescott T, Bull SS. Usability and navigability of an HIV/AIDS internet intervention for adolescents in a resource-limited setting. Comput Inform Nurs. 2012 Nov;30(11):587-95; quiz 596-7. PMID: 22918136. DOI: 10.1097/NXN.0b013e318266cb0e.

9. Ybarra ML, Bull SS, Prescott TL, Birungi R. Acceptability and feasibility of CyberSenga: an Internet-based HIV-prevention program for adolescents in Mbarara, Uganda. AIDS Care. 2014 Apr;26(4):441-7. PMID: 24093828. DOI: 10.1080/09540121.2013.841837.

10. Ybarra ML, Bull SS, Prescott TL, Korchmaros JD, Bangsberg DR, Kiwanuka JP. Adolescent abstinence and unprotected sex in CyberSenga, an Internet-based HIV prevention program: randomized clinical trial of efficacy. PLoS One. 2013 Aug 14;8(8):e70083. PMID: 23967069. DOI: 10.1371/journal.pone.0070083.

11. Gold J, Pedrana AE, Stoove MA, Chang S, Howard S, Asselin J, et al. Developing Health Promotion Interventions on Social Networking Sites: Recommendations from The FaceSpace Project. J Med Internet Res. 2012 Feb 28;14(1):e30. PMID: 22374589. DOI: 10.2196/jmir.1875.

12. Nguyen P, Gold J, Pedrana A, Chang S, Howard S, Ilic O, et al. Sexual health promotion on social networking sites: a process evaluation of The FaceSpace Project. J Adolesc Health. 2013 Jul;53(1):98-104. PMID: 23583509. DOI: 10.1016/j.jadohealth.2013.02.007.

13. van Rosmalen-Nooijens KAWL, Prins JB, Vergeer M, Wong SHLF, Lagro-Janssen ALM. « Young people, adult worries »: RCT of an internet-based self-support method « Feel the ViBe » for children, adolescents and young adults exposed to family violence, a study protocol. BMC Public Health. 2013 Mar 15;13:226. PMID: 23497359. DOI: 10.1186/1471-2458-13-226.

14. Winterling J, Wiklander M, Obol CM, Lampic C, Eriksson LE, Pelters B, et al. Development of a Self-Help Web-Based Intervention Targeting Young Cancer Patients With Sexual Problems and Fertility Distress in Collaboration With Patient Research Partners. JMIR Res Protoc. 2016 Apr 12;5(2):e60. PMID: 27073057. DOI: 10.2196/resprot.5499.

15. Mak DB, Bastian L, Grace J, Aquilina H, Sweeting J. Evaluation of a sexual health and blood-borne virus health education website for youth. Health Promot J Austr. 2012 Dec;23(3):194-200. PMID: 23540319

16. Rice E, Tulbert E, Cederbaum J, Barman Adhikari A, Milburn NG. Mobilizing homeless youth for HIV prevention: a social network analysis of the acceptability of a face-to-face and online social networking intervention. Health Educ Res. 2012 Apr;27(2):226-36. PMID: 22247453. DOI: 10.1093/her/cyr113.

17. Hightow-Weidman LB, Pike E, Fowler B, Matthews DM, Kibe J, McCoy R, et al. HealthMpowerment.org: feasibility and acceptability of delivering an internet intervention to young Black men who have sex with men. AIDS Care. 2012;24(7):910-20. PMID: 22272759. DOI: 10.1080/09540121.2011.647677.

18. Hightow-Weidman LB, LeGrand S, Muessig KE, Simmons RA, Soni K, Choi SK, et al. A Randomized Trial of an Online Risk Reduction Intervention for Young Black MSM. AIDS Behav. 2019 May;23(5):1166-1177. PMID: 30269231. DOI: 10.1007/s10461-018-2289-9

19. Bauermeister JA, Muessig KE, LeGrand S, Flores DD, Choi SK, Dong W, et al. HIV and Sexuality Stigma Reduction Through Engagement in Online Forums: Results from the HealthMPowerment Intervention. AIDS Behav. 2019 Mar;23(3):742-752. PMID: 30121727. DOI: 10.1007/s10461-018-2256-5.

20. Hightow-Weidman LB, Fowler B, Kibe J, McCoy R, Pike E, Calabria M, et al. HealthMpowerment.org: development of a theory-based HIV/STI website for young black MSM. AIDS Educ Prev. 2011 Feb;23(1):1-12. PMID: 21341956. DOI: 10.1521/aeap.2011.23.1.1.

21. Barry MC, Threats M, Blackburn NA, LeGrand S, Dong W, Pulley DV, Sallabank G, Harper GW, Hightow-Weidman LB, Bauermeister JA, Muessig KE. « Stay strong! keep ya head up! move on! it gets better!!!! »: resilience processes in the healthMpowerment online intervention of young black gay, bisexual and other men who have sex with men. AIDS Care. 2018 Aug;30(sup5):S27-S38. PMID: 30632775. DOI: 10.1080/09540121.2018.1510106.

22. Williams M, Bowen A, Ei S. An evaluation of the experiences of rural MSM who accessed an online HIV/AIDS health promotion intervention. Health Promot Pract. 2010 Jul;11(4):474-82. PMID: 19116419. DOI: 10.1177/1524839908324783.

23. O’Donnell NH, Willoughby JF. Photo-sharing social media for eHealth: analysing perceived message effectiveness of sexual health information on Instagram. J Vis Commun Med. 2017 Oct;40(4):149-159. PMID: 29022412. DOI: 10.1080/17453054.2017.1384995.

24. Ko N-Y, Hsieh C-H, Wang M-C, Lee C, Chen C-L, Chung A-C, et al. Effects of Internet popular opinion leaders (iPOL) among Internet-using men who have sex with men. J Med Internet Res. 2013 Feb 25;15(2):e40. PMID: 23439583. DOI: 10.2196/jmir.2264.

25. Villegas N, Santisteban D, Cianelli R, Ferrer L, Ambrosia T, Peragallo N, et al. Pilot testing an internet-based STI and HIV prevention intervention with Chilean women. J Nurs Scholarsh. 2015 Mar;47(2):106-16. PMID: 25410132. DOI: 10.1111/jnu.12114.

26. Villegas N, Santisteban D, Cianelli R, Ferrer L, Ambrosia T, Peragallo N, et al. The development, feasibility and acceptability of an Internet-based STI-HIV prevention intervention for young Chilean women. Int Nurs Rev. 2014 Mar;61(1):55-63. PMID: 24512261. DOI: 10.1111/inr.12080.

27. Bull SS, Levine DK, Black SR, Schmiege SJ, Santelli J. Research article: Social Media–Delivered Sexual Health Intervention. A Cluster Randomized Controlled Trial. Am J Prev Med. 2012 Nov;43(5):467-74. PMID: 23079168. DOI: 10.1016/j.amepre.2012.07.022.

28. Bull SS, Breslin LT, Wright EE, Black SR, Levine D, Santelli JS. Case study: An ethics case study of HIV prevention research on Facebook: the Just/Us study. J Pediatr Psychol. 2011 Nov-Dec;36(10):1082-92. PMID: 21292724. DOI: 10.1093/jpepsy/jsq126.

29. Bull SS, Levine D, Schmiege S, Santelli J. Recruitment and retention of youth for research using social media: Experiences from the Just/Us study. Vulnerable Child Youth Stud. 1 juin 2013;8(2):171‑81.

30. Greene GJ, Madkins K, Andrews K, Dispenza J, Mustanski B. Implementation and Evaluation of the Keep It Up! Online HIV Prevention Intervention in a Community-Based Setting. AIDS Educ Prev. 2016 Jun;28(3):231-45. PMID: 27244191. DOI: 10.1521/aeap.2016.28.3.231.

31. Mustanski B, Garofalo R, Monahan C, Gratzer B, Andrews R. Feasibility, acceptability, and preliminary efficacy of an online HIV prevention program for diverse young men who have sex with men: the keep it up! intervention. AIDS Behav. 2013 Nov;17(9):2999-3012. PMID: 23673793. DOI: 10.1007/s10461-013-0507-z.

32. Mustanski B, Parsons JT, Sullivan PS, Madkins K, Rosenberg E, Swann G. Biomedical and Behavioral Outcomes of Keep It Up!: An eHealth HIV Prevention Program RCT. Am J Prev Med. 2018 Aug;55(2):151-158. PMID: 29937115. DOI: 10.1016/j.amepre.2018.04.026.

33. Motley DN, Hammond S, Mustanski B. Strategies Chosen by YMSM During Goal Setting to Reduce Risk for HIV and Other Sexually Transmitted Infections: Results From the Keep It Up! 2.0 Prevention Trial. AIDS Educ Prev. 2017 Feb;29(1):1-13. PMID: 28195780. DOI: 10.1521/aeap.2017.29.1.1.

34. Mustanski B, Madkins K, Greene GJ, Parsons JT, Johnson BA, Sullivan P, et al. Internet-Based HIV Prevention With At-Home Sexually Transmitted Infection Testing for Young Men Having Sex With Men: Study Protocol of a Randomized Controlled Trial of Keep It Up! 2.0. JMIR Res Protoc. 2017 Jan 7;6(1):e1. PMID: 28062389. DOI: 10.2196/resprot.5740.

35. Gilliam M, Jagoda P, Jaworski E, Hebert LE, Lyman P, Wilson MC. « Because if We Don’t Talk about It, How Are We Going to Prevent It? »: « Lucidity, » a Narrative-Based Digital Game about Sexual Violence. Sex Educ Sex Soc Learn. 1 janv 2016;16(4):391‑404.

36. Scull TM, Kupersmidt JB, Malik CV, Keefe EM. Examining the Efficacy of an mHealth Media Literacy Education Program for Sexual Health Promotion in Older Adolescents Attending Community College. J Am Coll Health. 2018 Apr;66(3):165-177. PMID: 29068772. DOI: 10.1080/07448481.2017.1393822.

37. Campo S, Askelson NM, Spies EL, Losch M. Caution, the Use of Humor May Lead to Confusion: Evaluation of a Video Podcast of the Midwest Teen Sex Show. Am J Sex Educ. 2010 jan 1;5(3):201‑16.

38. Chen E, Barrington C. « You Can Do It Anywhere »: Student and Teacher Perceptions of an Online Sexuality Education Intervention. Am J Sex Educ. 2017 jan 1;12(2):105‑19.

39. Payton FC, Kvasny L. Online HIV awareness and technology affordance benefits for black female collegians - maybe not: the case of stigma. J Am Med Inform Assoc. 2016 Nov;23(6):1121-1126. PMID: 27094988. DOI: 10.1093/jamia/ocw017.

40. Glass N, Clough A, Case J, Hanson G, Barnes-Hoyt J, Waterbury A, et al. A safety app to respond to dating violence for college women and their friends: the MyPlan study randomized controlled trial protocol. BMC Public Health. 2015 Sep 8;15:871. PMID: 26350482. DOI: 10.1186/s12889-015-2191-6.

41. Alhusen J, Bloom T, Clough A, Glass N. Development of the MyPlan Safety Decision App with Friends of College Women in Abusive Dating Relationships. J Technol Hum Serv. 2015 July ;33(3):263‑82.

42. Lindsay M, Messing JT, Thaller J, Baldwin A, Clough A, Bloom T, et al. Survivor Feedback on a Safety Decision Aid Smartphone Application for College-Age Women in Abusive Relationships. J Technol Hum Serv. 2013oct; 31(4):368‑88.

43. Draper JL. SaVE Our Campus: Analyzing the Effectiveness of an Online Sexual Violence Program [Internet]. ProQuest LLC; 2017. Avalable on: https://rucore.libraries.rutgers.edu/rutgers-lib/52046/PDF/1/play/

44. Oliveira RNG de, Gessner R, Souza V de, Fonseca RMGS da. Limites e possibilidades de um jogo online para a construção de conhecimento de adolescentes sobre a sexualidade / Limits and possibilities of an online game for building adolescents’ knowledge of sexuality. Cien Saude Colet. 2016 Aug;21(8):2383-92. PMID: 27557012. DOI: 10.1590/1413-81232015218.04572016.

45. Souza V de, Gazzinelli MF, Soares AN, Fernandes MM, Oliveira RNG de, Fonseca RMGS da. The game as strategy for approach to sexuality with adolescents: theoretical-methodological reﬂections. Rev Bras Enferm. 2017 Apr;70(2):376-383. PMID: 28403303. DOI: 10.1590/0034-7167-2016-0043.

46. Fiellin LE, Kyriakides TC, Hieftje KD, Pendergrass TM, Duncan LR, Dziura JD, et al. The design and implementation of a randomized controlled trial of a risk reduction and human immunodeficiency virus prevention videogame intervention in minority adolescents: PlayForward: Elm City Stories. Clin Trials. 2016 Aug;13(4):400-8. PMID: 27013483. DOI: 10.1177/1740774516637871.

47. Hieftje K, Fiellin LE, Pendergrass T, Duncan LR. Development of an HIV Prevention Videogame Intervention: Lessons Learned. Int J Serious Games. 2016;3:83–90.

48. Duncan LR, Hieftje KD, Culyba S, Fiellin LE. Game playbooks: tools to guide multidisciplinary teams in developing videogame-based behavior change interventions. Transl Behav Med. 2014 Mar;4(1):108-16. PMID: 24653781. DOI: 10.1007/s13142-013-0246-8.

49. Mustanski B, Greene GJ, Ryan D, Whitton SW. Feasibility, acceptability, and initial efficacy of an online sexual health promotion program for LGBT youth: the Queer Sex Ed intervention. J Sex Res. 2015;52(2):220-30. PMID: 24588408. DOI: 10.1080/00224499.2013.867924.

50. Yeo TED, Chu TH. Sharing « Sex Secrets » on Facebook: A Content Analysis of Youth Peer Communication and Advice Exchange on Social Media about Sexual Health and Intimate Relations. J Health Commun. 2017 Sep;22(9):753-762. PMID: 28796578. DOI: 10.1080/10810730.2017.1347217.

51. Nicholas A, Murray E, Bailey JV, Stevenson F. The Sexunzipped trial: young people’s views of participating in an online randomized controlled trial. J Med Internet Res. 2013 Dec 12;15(12):e276. PMID: 24334198. DOI: 10.2196/jmir.2647

52. Bailey JV, Pavlou M, Copas A, McCarthy O, Carswell K, Rait G, et al. The Sexunzipped trial: optimizing the design of online randomized controlled trials. J Med Internet Res. 2013 Dec 11;15(12):e278. PMID: 24334216. DOI: 10.2196/jmir.2668.

53. Bailey J, Carswell K, Murray E, McCarthy O. Integrating psychological theory into the design of an online intervention for sexual health: the sexunzipped website. JMIR Res Protoc. 2012 Nov 19;1(2):e16. PMID: 23612122. DOI: 10.2196/resprot.2114.

54. McCarthy O, Carswell K, Murray E, Free C, Stevenson F, Bailey JV. What young people want from a sexual health website: design and development of Sexunzipped. J Med Internet Res. 2012 Oct 12;14(5):e127. PMID: 23060424. DOI: 10.2196/jmir.2116.

55. Danielson CK, McCauley JL, Jones A, Borkman AO, Miller S, Ruggiero KJ. Feasibility of Delivering Evidence-Based HIV/STI Prevention Programming to A Community Sample of African-American Teen Girls via the Internet. AIDS Educ Prev. 2013 Oct;25(5):394-404. PMID: 24059877. DOI: 10.1521/aeap.2013.25.5.394.

56. Nielsen A, De Costa A, Bågenholm A, Danielsson KG, Marrone G, Boman J, Salazar M, Diwan V. Trial protocol: a parallel group, individually randomized clinical trial to evaluate the effect of a mobile phone application to improve sexual health among youth in Stockholm County. BMC Public Health. 2018 Feb 5;18(1):216. PMID: 29402241. DOI: 10.1186/s12889-018-5110-9.

57. Mejia CM, Acland D, Buzdugan R, Grimball R, Natoli L, McGrath MR, et al. An Intervention Using Gamification to Increase Human Immunodeficiency Virus and Sexually Transmitted Infection Screening Among Young Men Who Have Sex With Men in California: Rationale and Design of Stick To It. JMIR Res Protoc. 2017 Jul 17;6(7):e140. PMID: 28716771. DOI: 10.2196/resprot.8064.

58. Brady SS, Sieving RE, Terveen LG, Rosser BRS, Kodet AJ, Rothberg VD. An Interactive Website to Reduce Sexual Risk Behavior: Process Evaluation of TeensTalkHealth. JMIR Res Protoc. 2015 Sep 2;4(3):e106. PMID: 26336157. DOI: 10.2196/resprot.3440.

59. Zhang Q, Huhn KJ, Tan A, Douglas RE, Li HG, Murti M, et al. « Testing is Healthy » TimePlay campaign: Evaluation of sexual health promotion gamification intervention targeting young adults. Can J Public Health. 2017 Apr 20;108(1):e85-e90. PMID: 28425904. DOI: 10.17269/cjph.108.5634.

60. Tanner AE, Mann L, Song E, Alonzo J, Schafer K, Arellano E, et al. weCARE: A Social Media-Based Intervention Designed to Increase HIV Care Linkage, Retention, and Health Outcomes for Racially and Ethnically Diverse Young MSM. AIDS Educ Prev. 2016 Jun;28(3):216-30. PMID: 27244190. DOI: 10.1521/aeap.2016.28.3.216.
