# Supplementary material for: Participatory Interventions for Sexual Health Promotion for Adolescents and Young Adults on the Internet: Systematic Review
Source: J Med Internet Res. 2020 Jul 31;22(7):e15378. doi: 10.2196/15378 (PMC7428916; doi:10.2196/15378)
Supplement: Multimedia Appendix 6 [file jmir_v22i7e15378_app6.docx]

**Multimedia Appendix 5 : Methodology intervention**

| **Name** | **Study included** | **Protocol only** | **Health study** | **Proc. study** | **n=** | **Year** | **Behavior change theory** | **Study design** | **Control group** | **Recruitment** | **Incentives** | **Health outcomes evaluated or planned** | **Follow-up (months)** | **Process outcomes evaluated** |
| --- | --- | --- | --- | --- | --- | --- | --- | --- | --- | --- | --- | --- | --- | --- |
| **+CLICK** | Markham CM, 2009 |  | X | X | 32 | 2007 | Social Cognitive Theory, self-regulation theory, Brief Motivational Enhancement Therapy | Pre-/post-test study | / | Clinics, community organizations | NR | Condom use self-efficacy ; positive trends toward importance; waiting before having sex ***(evaluated)*** | 3 | Feasibility, Acceptability, Usability |
| **No name** | Lou CH, 2006 |  | X | X | 1337 | 2003 | NR | RCT | NR | Community organizations | NR | Overall knowledge and of each specific aspect of reproductive health, attitudes and behaviors  ***(evaluated)*** | 10 | Attractiveness |
| **No name** | Sun WH, 2017 |  | X | X | 194 | 2016 | IMB model | RCT | Website directed by experts other than peers | SNS, e-mail, students organization | Direct remuneration | Condom use attitude, behavioral skills ***(evaluated)*** | 3 | Attractiveness, online experiences |
| **No name** | Widman L, 2016 |  | X |  | 300 | NR | NR | Exp. plan, Pre-/post-test study | / | Schools | Direct remuneration | Pubertal development, sexual outcome expectancies, hypothetical scenarios, sexual history, (Sexual activity/behavior), peer influence susceptibility ***(evaluated)*** | 0 | Not evaluated |
| **No name** | Fitzpatrick, 2018 | X |  |  | NR | 2018 | NR | RCT | Control arm view no materials and will not be invited to submit suggestions | Smartphone applications | Direct remuneration | HBsAg and anti-HCV IgG test uptake confirmed by test report photo at 4 weeks post-enrolment ***(planned)*** | 1 | Not evaluated |
| **No name** | Ybarra ML, 2014 |  | X | X | 75 | 2012-2013 | NR | Cross-sectional study | / | Community organizations | Gift card | Behavioral/ attitudinal change ***(evaluated)*** | 3 | Attractiveness, feedback |
| **CyberSenga** | Ybarra ML, 2012 |  |  | X | 20 | 2011 | IMB model | RCT | Nothing | Schools | No | 1) sexual abstinence; and 2) condom use during vaginal sex ***(evaluated)*** | 6 | Feasibility, Acceptability, Attractiveness, program navigation |
|  | Ybarra ML, 2014 |  |  | X | 366 |  |  |  |  |  |  |  |  |  |
|  | Bull S, 2010 |  |  | X | NR |  |  |  |  |  |  |  |  |  |
|  | Ybarra ML, 2013 |  | X |  | 366 |  |  |  |  |  |  |  |  |  |
| **FaceSpace project** | Nguyen P, 2013 |  |  | X | 173 | 2009-2010 | NR | Cross-sectional survey | / | SNS, e-mail | Book or movie voucher | Sexual risk and health-seeking behavior ***(planned)*** | NR | Reach project; Engagement |
|  | Gold J, 2012 |  |  | X | NR |  |  |  |  |  |  |  |  |  |
| **Feel The Vibe** | van Rosmalen-Nooijens KA, 2013 | X |  |  | NR | 2011 | NR | RCT | Usual care minimally improved | Internet, Social Network Sites | NR | PTSD symptoms, symptoms of depression and anxiety, Knowledge on sexual and reproductive health ***(planned)*** | 6 | Not evaluated |
| **Fex-Can Project** | Winterling J, 2016 | X |  |  | NR | NR | NR | Conception study | / | Registers | NR | Fertility distress ***(planned)*** | NR | Not evaluated |
| **Get the Facts** | Mak DB, 2012 |  |  | X | 491 | 2009-2010 | NR | Cross sectionnal study | / | NR | NR | NR | 18 | Attractiveness, Website usage |
| **Have You Heard** | Rice E, 2012 |  |  | X | 163 | 2009 | Social identity theory and theories of community mobilization and empowerment via participatory community theater models | Pilot study | / | Community organizations, by peers, internet | Direct remuneration (only for peer leaders) | NR | No | Acceptability |
| **HealthMpowerment** | Bauermeister JA, 2018 |  | X |  | 238 | 2012-2016 | NR | RCT | Information-only control website | Internet, SNS, clinics, word-of-mouth, flyers | Direct remuneration | Internalized Homophobia, Sexual Prejudice, HIV Stigma ***(evaluated)*** | 12 | Feasibility, Acceptability, Satisfaction, Usage |
|  | Hightow-Weidman, 2012 |  |  | X | NR |  |  |  |  |  |  |  |  |  |
|  | Hightow-Weidman, 2012 |  | X | X | 50 |  |  |  |  |  |  |  |  |  |
|  | Barry MC, 2019 |  | X |  | 48 |  |  |  |  |  |  |  |  |  |
|  | Hightow-Weidman, 2018 |  | X | X | 474 |  |  |  |  |  |  |  |  |  |
| **HOPE** | Williams M, 2010 |  |  | X | 300 | NR | IMB model | NR | / | Internet | NR | NR | NR | Satisfaction |
| **In Case You're Curious (ICYC)** | O'Donnell NH, 2017 |  | X | X | 839 | NR | NR | RCT | 4 experimental conditions | Universities | NR | Perceived message effectiveness, Attitudes toward condoms, systematic processing of sexual health information, perceived message sensation value and exposure to experimental messages ***(evaluated)*** | No | Message design impact on perceived message effectiveness |
| **iPOL** | Ko NY, 2013 |  | X |  | 1037 | 2011 | NR | Quasi-experimental study | Information-only control website | Internet, Social Network Sites | No | HIV test history (date and result of the last test), recreational drug use, history of STIs, and sexual behavior ***(evaluated)*** | 3 | Not evaluated |
| **I-STIPI** | Villegas N, 2014 |  | X |  | 40 | NR | IMB model | Pretest‐post-test study | / | e-mail, phone | Direct remuneration | Change in STI‐ and HIV‐Related Information, Motivation, Behavioral Skills, Behaviors, and Intimate Partner Violence ***(evaluated)*** | 1 | Feasibility, Acceptability, Recommendations for improvement |
|  | Villegas N, 2015 |  |  | X | 40 |  |  |  |  |  |  |  |  |  |
| **Just/Us** | Bull SS, 2012, |  | X |  | 652 | 2010-2011 | NR | RCT | Facebook page other than SRH, Group news | Internet, SNS | Gift card | Sexual behaviors ***(evaluated)*** | 6 | Ethics considerations |
|  | Bull SS, 2013 |  |  | X | NR |  |  |  |  |  |  |  |  |  |
|  | Bull SS, 2011 |  |  | X | NR |  |  |  |  |  |  |  |  |  |
| **Keep It Up!** | Greene GJ, 2016, |  | X | X | 343 | 2012, 2013, 2015 | IMB model | RCT | Information-only control website | Internet, Social Network Sites, previous studies, clinics, street, community-based HIV testing organizations, local and national advertising | Direct remuneration, Gift card, 50$ raffle | Incidence STI (primary outcome) - Sexual Health and HIV Risk Behaviors, HIV Knowledge, Condom Errors, Intentions to Use Condoms,  Self-Efficacy,  Decisional Balance  ***(evaluated)*** | 12 | Acceptability |
|  | Mustanski B, 2017 | X |  |  | 901 |  |  |  |  |  |  |  |  |  |
|  | Mustanski B, 2018 |  | X |  | 901 |  |  |  |  |  |  |  |  |  |
|  | Mustanski B, 2013 |  | X | X | 102 |  |  |  |  |  |  |  |  |  |
|  | Motley DN, 2017 |  | X |  | 901 |  |  |  |  |  |  |  |  |  |
| **Lucidity** | Gilliam M, 2016 |  | X | X | 24 | 2012 | NR | Conception study | / | Secondary schools, youth programs | NR | Communication about sexual violence ***(evaluated)*** | 0,5 | Attractiveness |
| **Media Aware** | Scull TM, 2018 |  | X | X | 281 | 2015 | NR | RCT | Wait-list control group | Universities | Gift card | Sexual risk behaviors (primary outcome), attitudes, injunctive normative beliefs, self-efficacy, willingness, and intentions related to risky sexual activity, condom/contraception use, and SRH communication (secondary outcomes) ***(evaluated)*** | NR | Feasibility, Satisfaction, Fidelity of implementation |
| **Midwest Teen Sex Show (MTSS)** | Campo S, 2010 |  |  | X | 41 | NR | NR | Formative study | / | NR | NR | NR | NR | Attractiveness, value of using humor to attract and engage |
| **MyHealthEd** | Chen E, 2017 |  |  | X | 29 | 2015 | NR | Pilot study | / | Secondary schools | Gift card | NR | NR | Attractiveness, Acceptability |
| **myHealthImpactNetwork** | Payton FC, 2016 |  |  | X | 60 | NR | Affordance theory | NR | / | NR | Gift card | NR | NR | Acceptability |
| **MyPlan** | Glass N, 2015 | X |  |  | 300 | NR | Dutton’s empowerment model | RCT | Generic Information Application | Internet, Social Network Sites, Universities, word-of mouth, phone | Direct remuneration, Gift card | Use of safety strategies, Decisional conflict ***(planned)*** | 12 | Attractiveness, Acceptability, usefulness, understandability, appropriateness |
|  | Alhusen J, 2015 |  |  | X | 31 |  |  |  |  |  |  |  |  |  |
|  | Lindsay M, 2013 |  |  | X | 38 |  |  |  |  |  |  |  |  |  |
| **Not Anymore** | Draper JL, 2017 |  | X |  | 2522 | NR | Cognitive Dissonance Theory | Pretest-post-test design | / | Universities | Gift card | Knowledge, attitudes around rape myths changed, and retained information ***(evaluated)*** | 3 | Not evaluated |
| **Papo Reto** | Oliveira RNG de, 2016, |  |  | X | 23 | 2014-2015 | NR | Exp. plan | / | Secondary schools | NR | Knowledge ***(planned)*** | 3 | Attractiveness, Interactivity |
|  | Souza V de, 2017 |  |  | X | NR |  |  |  |  |  |  |  |  |  |
| **Play Forward: Elm City Stories** | Fiellin LE, 2016 |  |  | X | 333 | 2016 | Social learning theory, self-efficacy, message framing, and delay discounting | RCT | Attention/time control with non-serious video games | Secondary schools | Gift card | Knowledge, intentions, self-efficacy and actual behaviors ***(planned)*** | 24 | Implementation |
|  | Hieftje K, 2016 |  |  | X | NR |  |  |  |  |  |  |  |  |  |
|  | Duncan LR, 2014 | X |  |  | NR |  |  |  |  |  |  |  |  |  |
| **Queer Sex Ed** | Mustanski B, 2015 |  | X | X | 107 | 2012-2013 | IMB model | Pretest-post-test design | / | Social Network Sites | Direct remuneration | Sexual risk behavior ***(evaluated)*** | 5 | Feasibility, Acceptability |
| **Sex Secrets** | Yeo, 2017 |  |  | X | NR | 2015 | NR | Exploratory study | / | Social Network Sites | NR | Topic Types and Health Concerns, Expectations, Exploration | 6 | Utility (of peer resource on Facebook) |
| **Sexunzipped** | Bailey JV, 2013, |  |  | X | 1994 | 2010-2011 | Theory of reasoned action (TRA), Theory of planned behavior (TPB) | RCT | Information-only control website | Internet, Social Network Sites, e-mail, secondary schools, universities, by peers (word-of mouth), clinics, youth organizations | Direct remuneration, Gift card | Sexual behavior change (including sexual health knowledge, sexual communication self-efficacy, and intention) as well as sexual behavior (condom and contraception use, use of services, partner numbers), and self-reported sexually transmitted infections and pregnancy  ***(planned)*** | 3 | Feasibility, Attractiveness, Acceptability |
|  | Carswell K, 2012, | X |  |  | 902 |  |  |  |  |  |  |  |  |  |
|  | McCarthy O, 2012, |  |  | X | 67 |  |  |  |  |  |  |  |  |  |
|  | Nicholas A, 2013 |  |  | X | 22 |  |  |  |  |  |  |  |  |  |
| **Sihle Web** | Danielson CK, 2013 |  | X | X | 41 | NR | NR | Pre-/post-test study | / | Secondary schools, youth organizations | Direct remuneration | Sexual Behavior and Condom Use, Condom Self-efficacy, Partner Communication History, Self-esteem, Ethnic Pride ***(evaluated)*** | 12 | Feasibility |
| **Skyddslaget** | Nielsen A, 2018 | X |  |  | 268 | NR | NR | RCT | Fictitious application without interactive modules | Internet, clinics | NR | Condom use (primary), Number of partners, number of tests for C. trachomatis during the study period. Occurrence of pregnancy, occurrence of STI (secondary) ***(planned)*** | 6 | Not evaluated |
| **Stick to it!** | Mejia CM, 2017 | X |  |  | NR | 2017 | Self-determination theory | RCT | NR | Internet, Social Network Sites, clinics, videos, flyers | Points for lot | Repeat HIV/STI screening ***(planned)*** | 6 | Feasibility, Acceptability |
| **TeensTalkHealth** | Brady SS, 2015 |  |  | X | 147 | 2011 | IMB model | RCT | NR | Secondary schools, clinics | Direct remuneration | Condom use and other health behaviors, Motivation, SRH Knowledge ***(planned)*** | 4 | Feasibility, Acceptability |
| **Testing is Healthy (game: TimePlay)** | Zhang Q, 2017 |  |  | X | NR | 2014-2015 | NR | NR | / | NR | NR | NR | 12 | Implementation, process evaluation indicators |
| **weCare** | Tanner AE, 2016 | X |  |  | NR | 2016 | Social cognitive theory, theory of empowerment education | Comparative before/after design | / | Health Educator | NR | HIV-related care engagement, health outcomes, ART prescription and adherence, viral suppression ***(planned)*** | 12 | Not evaluated |
